# Deregulation of ZPR1 causes respiratory failure in spinal muscular atrophy

**DOI:** 10.1038/s41598-017-07603-z

**Published:** 2017-08-15

**Authors:** Naresh K. Genabai, Annapoorna Kannan, Saif Ahmad, Xiaoting Jiang, Kanchan Bhatia, Laxman Gangwani

**Affiliations:** 1 Center of Emphasis in Neurosciences, Texas Tech University Health Sciences Center, El Paso, TX 79905 USA; 2 Department of Biomedical Sciences, Paul L. Foster School of Medicine, Texas Tech University Health Sciences Center, El Paso, TX 79905 USA; 3 Graduate School of Biomedical Sciences, Texas Tech University Health Sciences Center, El Paso, TX 79905 USA

## Abstract

Spinal muscular atrophy (SMA) is caused by the low levels of survival motor neuron (SMN) protein and is characterized by motor neuron degeneration and muscle atrophy. Respiratory failure causes death in SMA but the underlying molecular mechanism is unknown. The zinc finger protein ZPR1 interacts with SMN. ZPR1 is down regulated in SMA patients. We report that ZPR1 functions downstream of SMN to regulate HoxA5 levels in phrenic motor neurons that control respiration. Spatiotemporal inactivation of *Zpr1* gene in motor neurons down-regulates HoxA5 and causes defects in the function of phrenic motor neurons that results in respiratory failure and perinatal lethality in mice. Modulation in ZPR1 levels directly correlates and influences levels of *HoxA5* transcription. In SMA mice, SMN-deficiency causes down-regulation of ZPR1 and HoxA5 that result in degeneration of phrenic motor neurons. Identification of ZPR1 and HoxA5 as potential targets provides a paradigm for developing strategies to treat respiratory distress in SMA.

## Introduction

Spinal muscular atrophy (SMA) is a neurodegenerative disease caused by mutation of the *survival of motor neuron 1* (*SMN1*) gene. In humans, a duplicated copy of the *SMN2* gene produces insufficient levels of full-length SMN protein due to alternative splicing that causes skipping of the last coding exon (exon7) and results in the truncated transcript^1^. The low levels of SMN result in degeneration of spinal motor neurons that causes muscle atrophy followed by symmetric limb paralysis and respiratory distress. Patients with severe SMA require ventilation for life support. Death of SMA patients is caused by respiratory failure^2, 3^. The molecular mechanism of respiratory failure caused by SMN deficiency in SMA is unknown. Currently, there is no treatment to prevent or alleviate respiratory distress in SMA.

ZPR1 is evolutionary conserved and ubiquitously expressed in mammalian cells^4–6^. ZPR1 binds to inactive receptor tyrosine kinases (RTKs), epidermal growth factor (EGF) and platelet-derived growth factor (PDGF) receptors, translocates from the cytoplasm to the nucleus in response to mitogen or serum treatment and is a component of downstream signaling of RTKs^4, 7^. The function of ZPR1 is unclear. Biochemical studies have shown that ZPR1 interacts with SMN protein and is required for accumulation of SMN in the sub-nuclear bodies, gems and Cajal bodies (CB)^8, 9^. The severity of SMA disease negatively correlates with the number of SMN containing nuclear bodies^10^. ZPR1 may contribute to the nuclear function of SMN in RNA biogenesis such as snRNP assembly^11^ and splicing^9^ associated with sub-nuclear bodies^12, 13^. Interaction of ZPR1 with SMN is disrupted in SMA patients that have *SMN1* mutations^9^. SMA patients express low levels of ZPR1. Reduced expression of *Zpr1* increases severity of SMA disease and *Zpr1* is a modifier of SMA^14, 15^. The low levels of ZPR1 in mice with SMA-like disease have been shown to increase the severity of disease and reduce the lifespan of SMA mice^14^.

Disruption of the *Zpr1* gene in mice results in embryonic lethality^8^. However, the function of ZPR1 in mammalian embryogenesis and development is unknown. In this study, we show that the *Zpr1* is critical for the development and function of the respiratory motor neuron system. Mutation of *Zpr1* in motor neurons during early embryogenesis causes progressive loss of phrenic motor neurons and impairs the function of phrenic nerve that result in diaphragmatic paralysis, and respiratory failure, and stillbirth of mice. Temporal analysis of ZPR1 function unraveled, (a) selective vulnerability of phrenic motor neurons to the low levels of ZPR1 and (b) sustained *Zpr1* gene activity is critical for the function of respiratory motor neurons. Molecular analysis shows that ZPR1 deficiency in motor neurons causes deregulation of *HoxA5* expression. Modulation in ZPR1 levels directly correlates and influences levels of *HoxA5* transcription. In SMA mice, SMN-deficiency causes deregulation of ZPR1 and HoxA5 and results in degeneration of phrenic motor neurons that regulate respiration^16^. Expression of HoxA5 is required for the normal development and function of the phrenic motor neuron system^17^. Results of genetic and molecular analysis suggest that the ZPR1 functions downstream of SMN to regulate HoxA5 levels in phrenic motor neurons. ZPR1 binds to *HoxA5* gene promoter and regulates its transcription. These findings provide insight into the molecular mechanism of respiratory failure in SMA and identify ZPR1 and HoxA5 as potential therapeutic targets for developing therapies to reduce the burden of respiratory distress in SMA.

## Results

### *Zpr1* mutation in motor neurons causes anatomical defects and respiratory failure

Reduced *Zpr1* gene dosage in mice has been shown to cause progressive loss of motor neurons, gait abnormalities and defects in the peripheral nervous system^18^. However, the function of ZPR1 in growth, differentiation and survival of motor neurons is unknown. To gain insight into the function of ZPR1, we created mice with a conditional *Zpr1* allele by flanking exon1 with *loxP* sites, *Zpr1* ^*F1*/*F1*^ (Supplementary Figure S1). To examine the function of ZPR1 in motor neurons, we generated mutant mice in which *Zpr1* was selectively mutated in motor neurons by crossing conditional *Zpr1* ^*F1*/*F1*^ mice with transgenic *Mnx1*-*Cre* (*Cre* driven by *Mnx1* or *Hlxb9* or *Hb9* promoter) mice. The *Hb9* gene expresses during early embryogenesis (E8.5–9.5) in the spinal cord motor neuron and required for consolidation of motor neuron identity during embryonic development^19^. Mutant mice with homozygous deletion of *Zpr1* exon 1 (*Zpr1* ^Δ1/Δ1^) in motor neurons, in the presence of transgene *Hb9*-*Cre*, are referred as (*Zpr1* ^*Hb9*MNΔ^).

**Figure 1:**
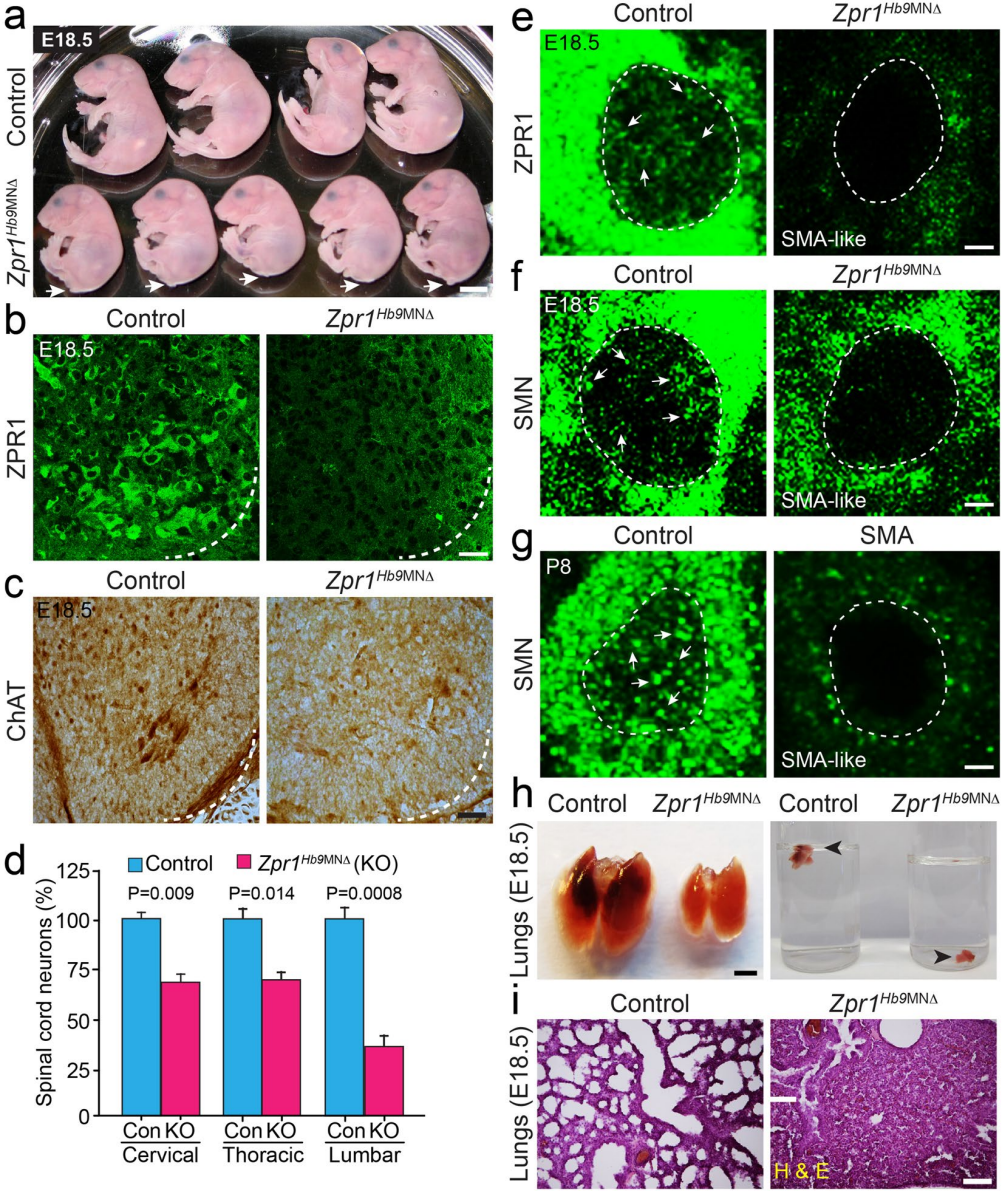
Mutation of the *Zpr1* gene in motor neurons causes developmental defects and respiratory failure in *Zpr1* ^*Hb9*MNΔ^ mice. (**a**) Photograph of E18.5 control and *Zpr1* ^*Hb9*MNΔ^ embryos showing developmental defects in mutant embryos, including smaller size and loss of tail. Arrows indicate the absence of tail in mutant embryos. All mutant *Zpr1* ^*Hb9*MNΔ^ mice were cyanotic and dead at birth (stillbirth). Scale bar is 5 mm. (**b**) Spinal cord sections from the thoracic region (T9-T12) of E18.5 control and *Zpr1* ^*Hb9*MNΔ^ embryos were stained with monoclonal antibody against murine ZPR1 (Clone LG-D5). *Zpr1* mutation in motor neurons results in reduced ZPR1 expression in spinal cord neurons of mutant embryos. Scale bar is 25 μm. (**c**) ZPR1 deficiency in motor neurons results in the loss spinal cord neurons. Spinal cord sections stained with antibody against choline acetyl transferase (ChAT). Scale bar is 100 μm. (**d**) Loss of neurons (mean ± s.e.m.; n = 3 mice/group) in different region of the spinal cords from *Zpr1* ^*Hb9*MNΔ^ embryos, cervical (31.96 ± 6.74%, p = 0.009), thoracic (30.52 ± 7.38%, p = 0.014) and lumbar (64.46 ± 7.10%, p = 0.0008) regions. (**e**) *Zpr1* mutation causes reduction in the cytoplasmic and nuclear staining of ZPR1 and (**f**) SMN in spinal cord neurons and show SMA-like phenotype. (**g**) Spinal cord motor neurons from SMA mice show phenotype similar to *Zpr1* ^*Hb9*MNΔ^ mice. Arrows indicate accumulation of ZPR1 and SMN in sub-nuclear bodies in control neurons but sub-nuclear bodies missing in *Zpr1* mutant and SMA mice. Dotted arcs represent the orientation of view from the anterior horn side of the spinal cord. Scale bar is 5 μm. (**h**) Lungs from E18.5 *Zpr1* ^*Hb9*MNΔ^ embryos were smaller than control and lungs of *Zpr1* ^*Hb9*MNΔ^ embryos immediately sank in water, indicating lungs were not inflated with air. Scale bar is 2.5 mm. (**i**) Histological analysis shows defects in morphogenesis of lungs from *Zpr1* ^*Hb9*MNΔ^ embryos and lungs were collapsed. Scale bar is 200 μm.

The *Zpr1* mutation in motor neurons causes defects in posterior axial growth and anatomical abnormalities. Mutant mice were born without tail, were cyanotic and found dead at birth (Fig. 1a). Anatomical defect suggested that there might also be defects in the skeleton development. Skeleton analysis of E18.5 embryos with X-ray (Supplementary Figure S2a,b) and staining with alcian blue and alizarin red shows that the skeleton is smaller but the skull of mutant (*Zpr1* ^*Hb9*MNΔ^) embryos is larger than control (Supplementary Figure S2c), however, the total numbers of vertebrae for the cervical, thoracic and lumbar regions of the spinal cord were same in mutant and control mice (Supplementary Figure S2d). Interestingly, skeleton defects noted in the sacral region, including underdeveloped ischium bones and fused sacral vertebrae (Supplementary Figure S2d–f) show similarity to congenital defects found in autosomal dominant Currarino syndrome (also known as hereditary sacral agenesis (HSA) or caudal regression syndrome) patients that have *HLXB9* mutation^20, 21^. Examination of thoracic rib cage showed smaller sternum with reduced ossification of lower sternebrae (Supplementary Figure S2g,h). However, the presence of anatomical and skeleton defects *Zpr1* ^*Hb9*MNΔ^ mice did not justify the severity that would lead to perinatal lethality. To determine whether death occurred *in utero* or at birth, we examined E18.5 embryos. Normal embryos were alive and started breathing, in contrast, rarely mutant embryos attempted to breathe but failed to initiate breathing and died within minutes (Supplementary Movie S1). This observation suggests that most of the mutant *Zpr1* ^*Hb9*MNΔ^ mice died *in utero* and respiratory failure may be the cause of death.

### ZPR1 deficiency causes degeneration of motor neurons and defects in the development of the respiratory system

To investigate the effect of ZPR1 deficiency on motor neurons, we examined the spinal cord motor neurons in control and *Zpr1* ^*Hb9*MNΔ^ mice. ZPR1 staining of the spinal cord sections show reduced expression of ZPR1 in *Zpr1* ^*Hb9*MNΔ^ mice compared to control mice (Fig. 1b, *quantitative analysis presented in* Fig. 8). Next, we examined the effect of ZPR1 deficiency on the number of motor neuron in different regions of the spinal cord. Spinal cord sections stained with choline acetyltransferase (ChAT) show reduced number of neurons in the ventral horns (Fig. 1c). Neuron count analysis shows the loss of neurons in the cervical (31.96 ± 6.74%, p = 0.009), thoracic (30.52 ± 7.38%, p = 0.014) and lumbar (64.46 ± 7.10%, p = 0.000) regions of the spinal cord from E18.5 *Zpr1* ^*Hb9*MNΔ^ embryos compared to control embryos (Fig. 1d), suggesting that ZPR1 is required for neuron growth and survival. Increased loss of neurons in the lumbar region supports anatomical defects, loss of tail and sacral agenesis, found in *Zpr1* ^*Hb9*MNΔ^ mice. Noticeably, the primary pathogenesis in SMA is the loss of motor neurons in the lumbar region. Further examination of motor neurons shows marked reduction in cytoplasmic and nuclear staining of ZPR1 and absence of ZPR1^+^ sub-nuclear bodies in neurons of mutant mice compared to control mice (Fig. 1e). ZPR1 deficiency causes reduced staining of SMN and the loss of SMN^+^ nuclear bodies in motor neurons and results in a SMA-like phenotype (Fig. 1f). Comparison of SMN stained motor neurons from *Zpr1* ^*Hb9*MNΔ^ and SMA mice (Fig. 1f,g) shows ZPR1 deficiency results in a phenotype similar to SMA.

To investigate the cause of death as respiratory failure in *Zpr1* ^*Hb9*MNΔ^ mice, first we examined lungs. The lungs from mutant *Zpr1* ^*Hb9*MNΔ^ mice were smaller compared to control and failed to float on the surface upon submerging into water (Fig. 1h), suggesting that lungs from mutant mice were not inflated with air. Histochemical staining of lungs from *Zpr1* ^*Hb9*MNΔ^ mice shows defects in lungs morphogenesis such as lack of alveolization (Fig. 1i). These findings suggest that the normal levels of ZPR1 in motor neurons are required for lungs development during mammalian embryogenesis. Similar defects in lungs development have been reported in mice with mutation of *Hox5* group of genes that result in respiratory failure and death of mice^17, 22, 23^. These findings indicate that the *Zpr1* and *Hox5* genes may be components of common pathway involved in the development of mammalian respiratory system. Lack of air in lungs suggests that diaphragmatic paralysis might be the cause of respiratory failure in *Zpr1* ^*Hb9*MNΔ^ mice.

### Defects in the phrenic nerve development and innervation of diaphragm in *Zpr1*^*Hb9*MNΔ^ mice

**Figure 2:**
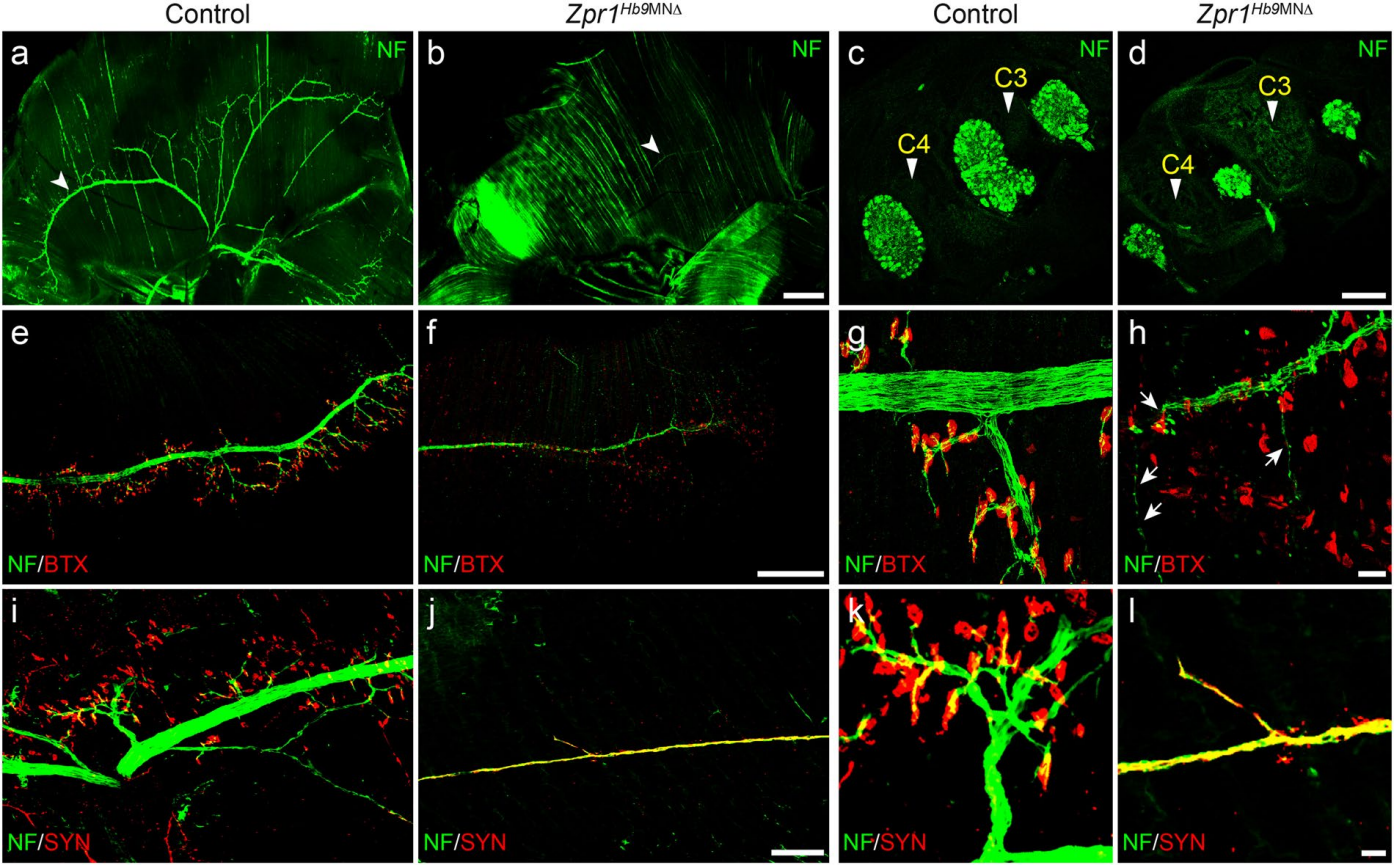
Defects in the development of phrenic nerve and diaphragm innervation in *Zpr1* ^*Hb9*MNΔ^ mice. (**a**,**b**) Whole diaphragms from E18.5 embryos were stained with neurofilament (NF) antibody. Images shown are composite of individual panels acquired by TileScan using confocal microscope. Immunofluorescence image analysis revealed defects in the development of phrenic nerve in *Zpr1* ^*Hb9*MNΔ^ embryos (arrowheads indicate phrenic nerve). (**c**,**d**) Analysis of longitudinal sections of spinal cord shows cross section of three bundles of respiratory motor neurons exiting from the C3-C5 region of the spinal cord that form phrenic nerve (arrowheads indicate cervical vertebrae). ZPR1 deficiency results in marked reduction in the size of all three bundles of motor axons, indicating ZPR1 is required for the development of phrenic motor neurons. (**e**,**h**) Analysis of diaphragms stained with NF and α-bungarotoxin (BTX) shows defects in the innervation of neuromuscular junctions (NMJs) by motor neurons in mutant embryos. The phrenic nerve reached diaphragm muscle but failed to generate secondary and tertiary branches and innervate NMJs in mutant embryos. Phrenic nerve degeneration was detected in mutant embryos (arrows). (**i**-**l**) Staining with NF and synaptophysin (SYN) show severe defects in the formation synapse in mutant embryos, a likely cause of stillbirth of *Zpr1* ^*Hb9*MNΔ^ mice. Scale bars are 500 μm (**a**,**b**), 250 μm (**c**,**d**,**e**,**f**), 100 μm (**i**,**j**) and 12.5 μm (**g**,**h**,**k**,**l**).

To further investigate the cause of respiratory failure, we examined the effect of *Zpr1* mutation on the development of phrenic nerves and innervation of the diaphragm. Diaphragms were stained with neurofilament (NF) and α-bungarotoxin (BTX) and NF and synaptophysin (SYN) from control and *Zpr1* ^*Hb9*MNΔ^ mice. Examination of whole mount diaphragm from E18.5 embryos shows severe defect in the development of phrenic nerves in *Zpr1* ^*Hb9*MNΔ^ mice. Defects included absence of secondary and tertiary branches; only a single primary branch was detected on one side of the diaphragm in most severe cases (Fig. 2a,b). Longitudinal spinal cord sections containing C3, C4 vertebrae show marked reduction in the diameter of three phrenic nerve branches exiting from the spinal cord in *Zpr1* ^*Hb9*MNΔ^ mice compared to control mice (Fig. 2c,d). Comparison of diameter of primary nerve in the diaphragm between control (21.44 ± 0.017 μm) and *Zpr1* ^*Hb9*MNΔ^ (5.88 ± 0.028 μm) shows marked reduction (72.23 ± 3.27%, p = 0.000) that is consistent with the loss of neurons in the cervical region of the spinal cord (Fig. 1d). Further, examination of diaphragm stained with NF and BTX shows severe defects in branching of phrenic nerve and innervation of neuromuscular junctions (NMJs) suggesting that the phrenic nerve was able to innervate diaphragm muscle but neurons failed to generate sufficient secondary and tertiary branches to innervate NMJs in *Zpr1* ^*Hb9*MNΔ^ mice (Fig. 2e–h). ZPR1 deficiency resulted in degeneration of secondary and tertiary nerve branches in mutant mice (Fig. 2h). Examination of neuromuscular synapses shows co-localization of NF and SYN in NMJs in control mice (Fig. 2i,k). In contrast, diaphragms from mutant *Zpr1* ^*Hb9*MNΔ^ mice showed a severe lack of synapse formation, SYN failed to localize to NMJs and was accumulated in primary branch of phrenic nerve (Fig. 2i–l). These data suggest that defects in synapse formation by phrenic nerve may be the cause of diaphragmatic paralysis that leads to respiratory failure and perinatal mortality in *Zpr1* ^*Hb9*MNΔ^ mice.

### ZPR1 deficiency causes progressive loss of phrenic motor neurons in *Zpr1*^*Hb9*MNΔ^ mice

**Figure 3:**
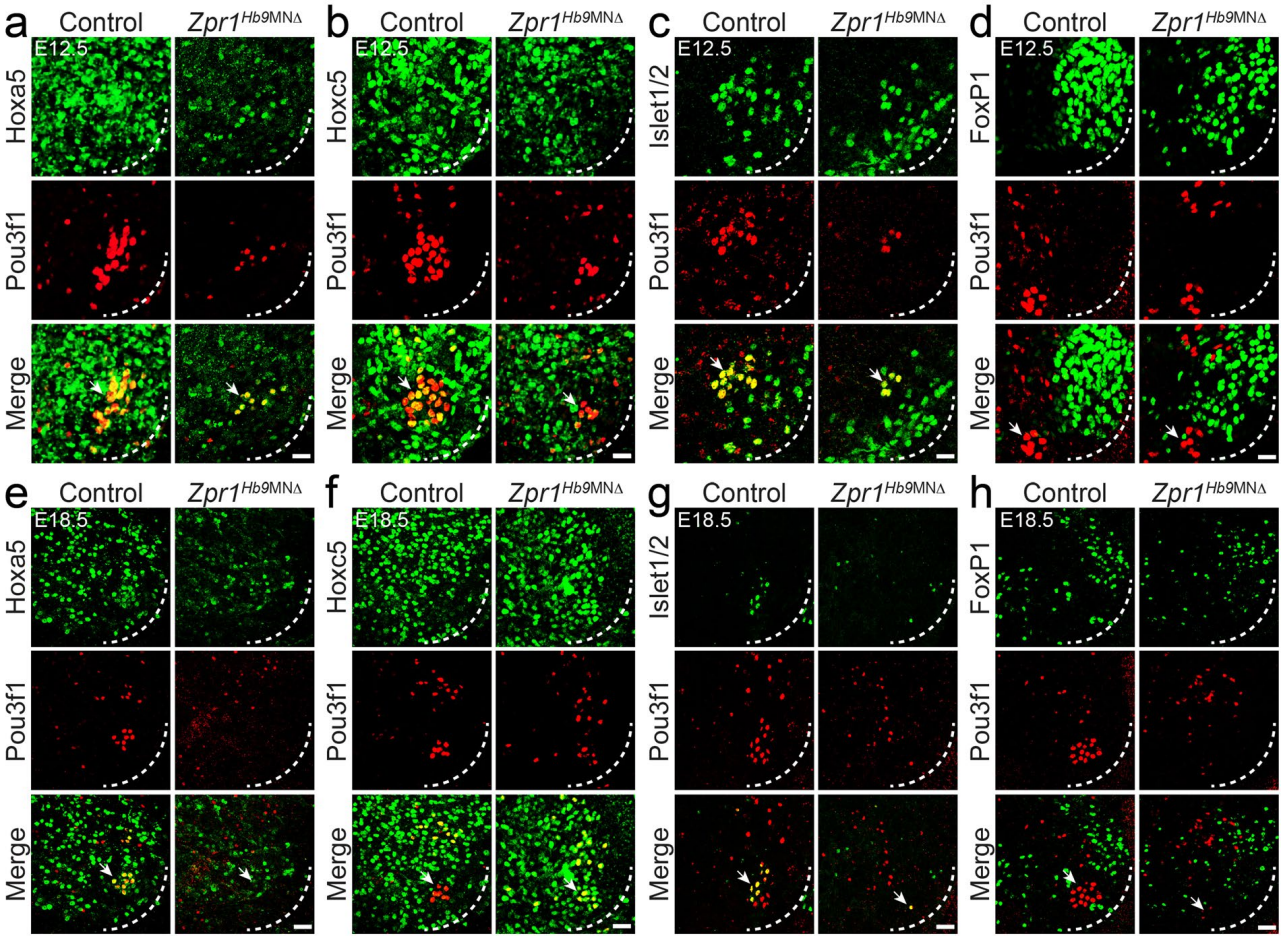
ZPR1 deficiency causes progressive loss of PMC neurons in *Zpr1* ^*Hb9*MNΔ^ mice. Spinal cord sections of the cervical region (C3-C5) from (**a**–**d**) E12.5 and (**e**–**h**) E18.5 control and *Zpr1* ^*Hb9*MNΔ^ embryos were stained with antibodies against Pou3f1, Hoxa5, Hoxc5, Islet1/2 and FoxP1 proteins. Immunofluorescence images reveal the effect of *Zpr1* mutation on the loss and disorganization of different populations of neurons in the cervical region, including PMC and LMC groups of motor neurons. Quantification of Pou3f1^+^, Hoxa5^+^, Hoxc5^+^, and FoxP1^+^ neurons at E12.5 and E18.5 stages is presented in Supplementary Figure S3. Neurons were counted in serial sections of C3-C5 region. Neuron losses are presented as (mean ± s.e.m.; n = 3 mice/group). At E12.5 stage, images (**a**–**d**) and quantification shows loss (64.29 ± 4.78%, p < 0.0001) of Pou3f1^+^ motor neurons. By E18.5 stage (**e**–**h**), Pou3f1^+^ neurons were barely detectable and quantification shows greater loss (90.00 ± 15.81%, p = 0.0013) of neurons in *Zpr1* ^*Hb9*MNΔ^ embryos. Image analysis and quantification of Hoxa5^+^ neurons shows non-progressive loss of neurons at E12.5 (64.64 ± 15.54%, p = 0.0032) and at E18.5 (59.35 ± 11.60%, p = 0.0022) stages in *Zpr1* ^*Hb9*MNΔ^ embryos. A smaller loss of Hoxc5^+^ neurons at E12.5 (31.93 ± 4.80%, p = 0.0002) and E18.5 (24.51 ± 9.81%, p = 0.0369) stages was noted in *Zpr1* ^*Hb9*MNΔ^ mice. A relatively smallest decrease in FoxP1^+^ LMC neurons at E12.5 (13.59 ± 10.94%, p = 0.2605) that increases to a statistically significant loss at E18.5 (28.17 ± 2.42%, p = 0.0001) was found in *Zpr1* ^*Hb9*MNΔ^ mice. Images (**d**,**h**) show disorganization and the loss of compactness of FoxP1^+^ LMC neurons. Dotted arcs represent the orientation of view from the anterior horn side of the spinal cord. Arrows indicate phrenic nerve motor neurons. Scale bars are 25 μm (**a**–**d**) and 50 μm (**e**–**h**).

Phrenic motor column (PMC) neurons are located between cervical C3-C5 segments and exit spinal cord to form phrenic nerves that innervate diaphragm^24^. Decrease in diameter of phrenic nerve in mutant compared to control mice suggests that ZPR1 deficiency might have caused loss of phrenic neurons (Fig. 2c,d). We examined the effect of *Zpr1* mutation on PMC neurons in *Zpr1* ^*Hb9*MNΔ^ mice. PMC neurons can be identified and distinguished from other neurons in cervical region using Pou3f1 (also known as Scip or Oct6), a POU-class transcription factor^25^, as a marker^17^. Pou3f1^+^ PMC neurons express high levels of Hoxa5, Hoxc5, Isl1 and Hb9^17^. Notably, Pou3f1^+^ PMC neurons do not express FoxP1^17^, a transcription factor required for *Hox* genes activity, and presence of FoxP1 allows distinguishing between limb-innervating or lateral motor column (LMC) neurons from other subtypes, including PMC neurons^26–28^. Double stained immunofluorescence images of the spinal cords from E12.5 (Fig. 3a–d) and E18.5 (Fig. 3e–h) control and mutant embryos show location of Pou3f1^+^ PMC neurons in the context of neurons stained positive for Hoxa5, Hoxc5, Islet1/2 and FoxP1. Quantitative analysis of Pou3f1^+^ PMC neurons at E12.5 stage shows *Zpr1* mutation causes loss (64.29 ± 4.78%, p = 0.000) of neurons in *Zpr1* ^*Hb9*MNΔ^ compared to control mice. At E18.5 stage the loss of Pou3f1^+^ neurons increases to (90.00 ± 15.81%, p = 0.001) and neurons were barely detectable in *Zpr1* ^*Hb9*MNΔ^ embryos, suggesting a progressive loss of PMC neurons between E12.5 to E18.5 stages of embryonic development (Supplementary Figure S3). *Zpr1* mutation also causes the loss of Hoxa5^+^, Hoxc5^+^ and FoxP1^+^ neurons during development (Fig. 3). However, the loss of Hoxa5^+^ neurons was non-progressive, E12.5 (64.64 ± 15.54%, p = 0.003) and E18.5 (59.35 ± 11.60%, p = 0.002). The loss of Hoxc5^+^ neurons is ~2-folds less than Hoxa5^+^ neurons and was also non-progressive, E12.5 (31.93 ± 4.80%, p = 0.000) and E18.5 (24.51 ± 9.81%, p = 0.036). Comparison of neuron loss shows highest loss of Pou3f1^+^ (~90%) followed by Hoxa5^+^ (~60%) and Hoxc5^+^ (~25%) neurons at E18.5 stage in *Zpr1* ^*Hb9*MNΔ^ mice. Comparison of average losses of Hoxa5^+^ neurons at E12.5 and E18.5 stages did not show significant change suggesting that ZPR1 is critical for the development and differentiation of Hoxa5^+^ neurons during early stages of embryogenesis. Notably, progressive loss of Pou3f1^+^ neurons between E12.5 and E18.5 stages suggests that sustained *Zpr1* gene activity is required for differentiation and maintenance PMC respiratory motor neurons throughout embryonic development.

*Zpr1* mutation results in disorganization of FoxP1^+^ neurons in *Zpr1* ^*Hb9*MNΔ^ embryos compared to control at E12.5 (Fig. 3d,h). Neuron counting did not show significant decrease in FoxP1^+^ neurons at E12.5 (13.59 ± 10.94%, p = 0.260). Interestingly, at E18.5 FoxP1^+^ neurons loss increased to (28.17 ± 2.42%, p = 0.0001) (Supplementary Figure S3d,h). To find whether ZPR1 deficient FoxP1^+^ neurons have limbs innervation defects similar to PMC neurons, we examined forelimbs and hindlimbs from E18.5 embryos stained with NF and BTX. Both forelimb and hindlimb muscles were innervated by motor neurons but reduced secondary and tertiary branches were noted in *Zpr1* ^*Hb9*MNΔ^ compared to control mice (Supplementary Figure S4). Defects were found in the innervation of NMJs in the hindlimb of *Zpr1* ^*Hb9*MNΔ^ mice. ZPR1 deficiency resulted in degeneration of secondary and tertiary nerve branches in forelimbs of *Zpr1* ^*Hb9*MNΔ^ mice. Comparison of innervation defect between diaphragm and limbs shows that limb innervation defects were less severe compared to diaphragm. These data suggest that the ZPR1 deficiency causes less severe defects in arborization of limb innervating motor neurons however ZPR1 is required for maintenance and function of terminal nerve branches.

### Temporal delay in *Zpr1* mutation preserves anatomical and skeleton development

To further investigate the role of ZPR1 in the functioning of the respiratory system, we examined the effect of temporal delay in *Zpr1* mutation in motor neurons using *choline acetlytransferase* (*ChAT*)-Cre mice. The *ChAT* gene expression begins shortly after differentiation of cervical motor neurons at mid-gestation embryonic stage ~E10.5–11.5 days. The *ChAT* driven Cre recombinase has been shown to effectively remove *floxed* gene from motor neurons by E13.5 stage^17^. We generated mutant mice in which *Zpr1* was selectively deleted from motor neurons by crossing conditional allele *Zpr1* ^*F1*/*F1*^ mice with transgenic *ChAT*-*Cre* mice. Mice with homozygous deletion of *Zpr1* exon 1 (*Zpr1* ^Δ1/Δ1^) in motor neurons, in the presence of transgene *ChAT*-*Cre*, are referred as (*Zpr1* ^*ChAT*MNΔ^).

**Figure 4:**
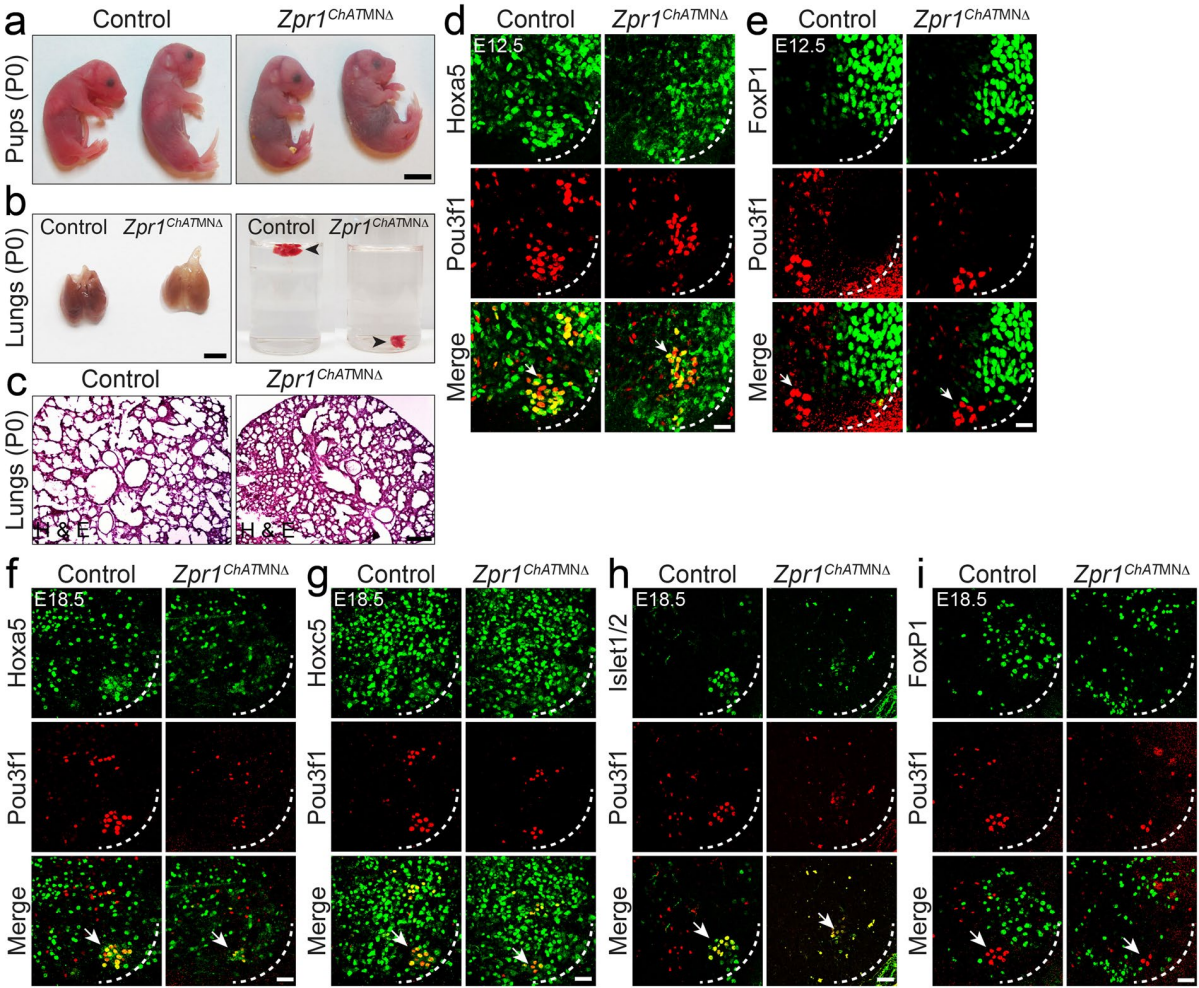
Temporal delay in mutation of *Zpr1* preserves anatomical development but causes loss of PMC neurons and respiratory failure in *Zpr1* ^*ChAT*MNΔ^ mice. (**a**) Photograph of control and *Zpr1* ^*ChAT*MNΔ^ pups (P0) showing normal anatomical development. All mutant *Zpr1* ^*ChAT*MNΔ^ mice (n > 20) were died immediately after birth. Scale bar is 5 mm. (**b**) Lungs from *Zpr1* ^*ChAT*MNΔ^ pups (P0) were smaller than control and lungs of mutant embryos immediately sank in water, indicating lungs were not inflated with air suggesting mutant mice failed to breathe after birth. Scale bar is 3 mm. (**c**) Histological analysis shows defects in morphogenesis of lungs from *Zpr1* ^*ChAT*MNΔ^ pups (P0) and lungs were underdeveloped. Scale bar is 200 μm. (**d**,**e**) Spinal cord sections of the cervical region (C3-C5) from E12.5 control and *Zpr1* ^*ChAT*MNΔ^ embryos were stained with antibodies against Pou3f1, Hoxa5 and FoxP1 proteins. Scale bar is 25 μm (**d**,**e**). Quantification of Pou3f1^+^, Hoxa5^+^ and FoxP1^+^ neurons (mean ± s.e.m.; n = 3 mice/group) at E12.5 stage is presented in Supplementary Figure S5a–c. (**f**–**i**) Spinal cord sections of the cervical region (C3-C5) from E18.5 control and *Zpr1* ^*ChAT*MNΔ^ embryos were stained with antibodies against Pou3f1, Hoxa5, Hoxc5, Islet1/2 and FoxP1 proteins. Immunofluorescence images show that the delayed *Zpr1* mutation causes loss of Pou3f1^+^ PMC motor neurons and disorganization of FoxP1^+^ LMC group of motor neurons. Quantification of Pou3f1^+^, Hoxa5^+^ and Hoxc5^+^ neurons at E18.5 stage is presented in Supplementary Figure S5d–f. Quantification of neurons shows the loss of Pou3f1^+^ neurons (61.90 ± 12.59%, p = 0.0026), Hoxa5^+^ neurons (48.34 ± 12.67%, p = 0.0189) and Hoxc5^+^ neurons (12.97 ± 1.93%, p = 0.0027) in E18.5 *Zpr1* ^*ChAT*MNΔ^ embryos. Scale bar is 50 μm (**d**–**i**).

Temporal delay in *Zpr1* mutation in motor neurons resulted in reduced severity and preserved anatomical and skeleton development in *Zpr1* ^*ChAT*MNΔ^ mice (Fig. 4a). Interestingly, mutant *Zpr1* ^*ChAT*MNΔ^ mice were born live but died within minutes of birth. This finding suggested that *Zpr1* ^*ChAT*MNΔ^ mice might have also died because of respiratory failure similar to *Zpr1* ^*Hb9*MNΔ^ mice. Therefore, first we examined the lungs from newborn control and *Zpr1* ^*ChAT*MNΔ^ mice. Lungs from mutant *Zpr1* ^*ChAT*MNΔ^ mice were smaller compared to control mice and failed to float to the surface upon submerging into water suggesting that the lungs were not inflated with air (Fig. 4b). Histological examination shows defects in the lungs morphogenesis in *Zpr1* ^*ChAT*MNΔ^ mice (Fig. 4c) however the severity of defect was less compared to *Zpr1* ^*Hb9*MNΔ^ mice (Fig. 1i). Together, findings from *Zpr1* ^*Hb9*MNΔ^ and *Zpr1* ^*ChAT*MNΔ^ mice suggest that the sustained expression of ZPR1 in motor neurons during E8.5-E13.5 window of embryogenesis is critical for the development of normal respiratory system, including lungs morphogenesis.

### Temporal delay in *Zpr1* mutation causes reduced loss of PMC neurons in *Zpr1*^*ChAT*MNΔ^ mice

The death of newborn *Zpr1* ^*ChAT*MNΔ^ mice due to respiratory failure might be a consequence of defects in the functioning of the phrenic nerve and diaphragm system. The delayed *Zpr1* mutation shows a smaller loss of Pou3f1^+^ (23.50 ± 9.77%, p = 0.037) and Hoxa5^+^ (35.56 ± 7.89%, p = 0.006) neurons (Fig. 4d,e and Supplementary Figure S5a–c) in *Zpr1* ^*ChAT*MNΔ^ compared to *Zpr1* ^*Hb9*MNΔ^, Pou3f1^+^ (64.29 ± 4.78%, p = 0.000) and Hoxa5^+^ (64.64 ± 15.54%, p = 0.003) in E12.5 embryos (Supplementary Figure S3). Interestingly, the loss of FoxP1^+^ (7.70 ± 7.89%, p = 0.1241) neurons was not significant at E12.5 stage suggesting that ZPR1 deficiency might have caused selective loss of Pou3f1^+^ and Hoxa5^+^ neurons at early stage due to delayed *Zpr1* mutation (Fig. 4d,e). Comparison of neuron loss at E12.5 stage between two mutant mice shows reduction in the loss of Pou3f1^+^ (~2.7-folds) and Hoxa5^+^ (~1.8-folds) neurons in *Zpr1* ^*ChAT*MNΔ^ mice compared to *Zpr1* ^*Hb9*MNΔ^ mice suggesting delayed *Zpr1* mutation preserves development and differentiation of PMC motor neurons. Analysis at E18.5 stage shows increased loss of Pou3f1^+^ (61.90 ± 12.59%, p = 0.002) and Hoxa5^+^ (48.34 ± 12.67%, p = 0.018) neurons in *Zpr1* ^*ChAT*MNΔ^ mice (Fig. 4f–i and Supplementary Figure S5d–f). Comparison of Pou3f1^+^ neuron loss at E12.5 and E18.5 stages shows increased (~2.6-fold) loss of Pou3f1^+^ neurons at E18.5 that suggest progressive loss of PMC neurons during development in *Zpr1* ^*ChAT*MNΔ^ mice that is consistent with *Zpr1* ^*Hb9*MNΔ^ mice (Supplementary Figures S3 and S5). The major loss of Pou3f1^+^ neurons among other neurons in both *Zpr1* ^*Hb9*MNΔ^ and *Zpr1* ^*ChAT*MNΔ^ mutant mice suggest that the PMC neurons are particularly sensitive to low levels of ZPR1 during development of the respiratory system. Comparison of neuron loss at E18.5 between two mutant mice shows that the late removal of *Zpr1* helps preserve ~28% more PMC neurons in *Zpr1* ^*ChAT*MNΔ^ than *Zpr1* ^*Hb9*MNΔ^ mice, however, *Zpr1* ^*ChAT*MNΔ^ mice failed to breathe after birth. These data suggest that the PMC neurons in *Zpr1* ^*ChAT*MNΔ^ mice might not have potential to perform respiratory function.

### Temporal delay in *Zpr1* mutation improves diaphragm innervation in *Zpr1*^*ChAT*MNΔ^ mice

**Figure 5:**
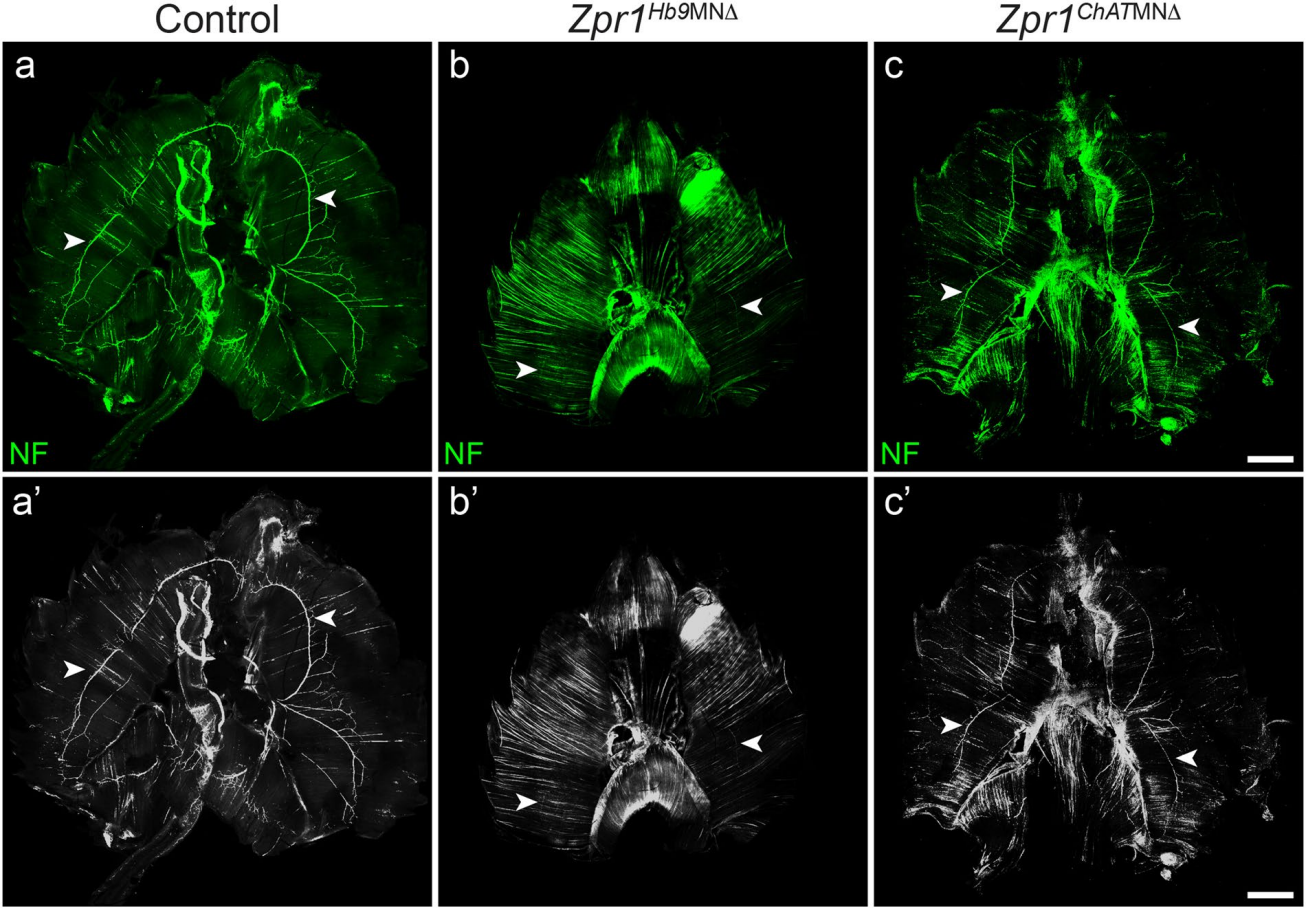
Temporal delay in *Zpr1* mutation reduces severity of defects in the development of respiratory system. Whole diaphragms from E18.5 embryos were stained with neurofilament (NF) antibody. Images shown are composite of individual panels acquired by TileScan using confocal microscope. Diaphragms from (**a**,a’) control (**b**,b’) *Zpr1* ^*Hb9*MNΔ^ and (**c**,c’) *Zpr1* ^*ChAT*MNΔ^ mice showing innervation of phrenic nerve in the muscle. Comparison of the phrenic nerve staining between *Zpr1* ^*Hb9*MNΔ^ and *Zpr1* ^*ChAT*MNΔ^ mice shows remarkable improvement in the development and innervation of phrenic nerve upon temporal delay in *Zpr1* mutation in motor neurons. Arrowheads indicate phrenic nerve. Scale bar is 1000 μm.

**Figure 6:**
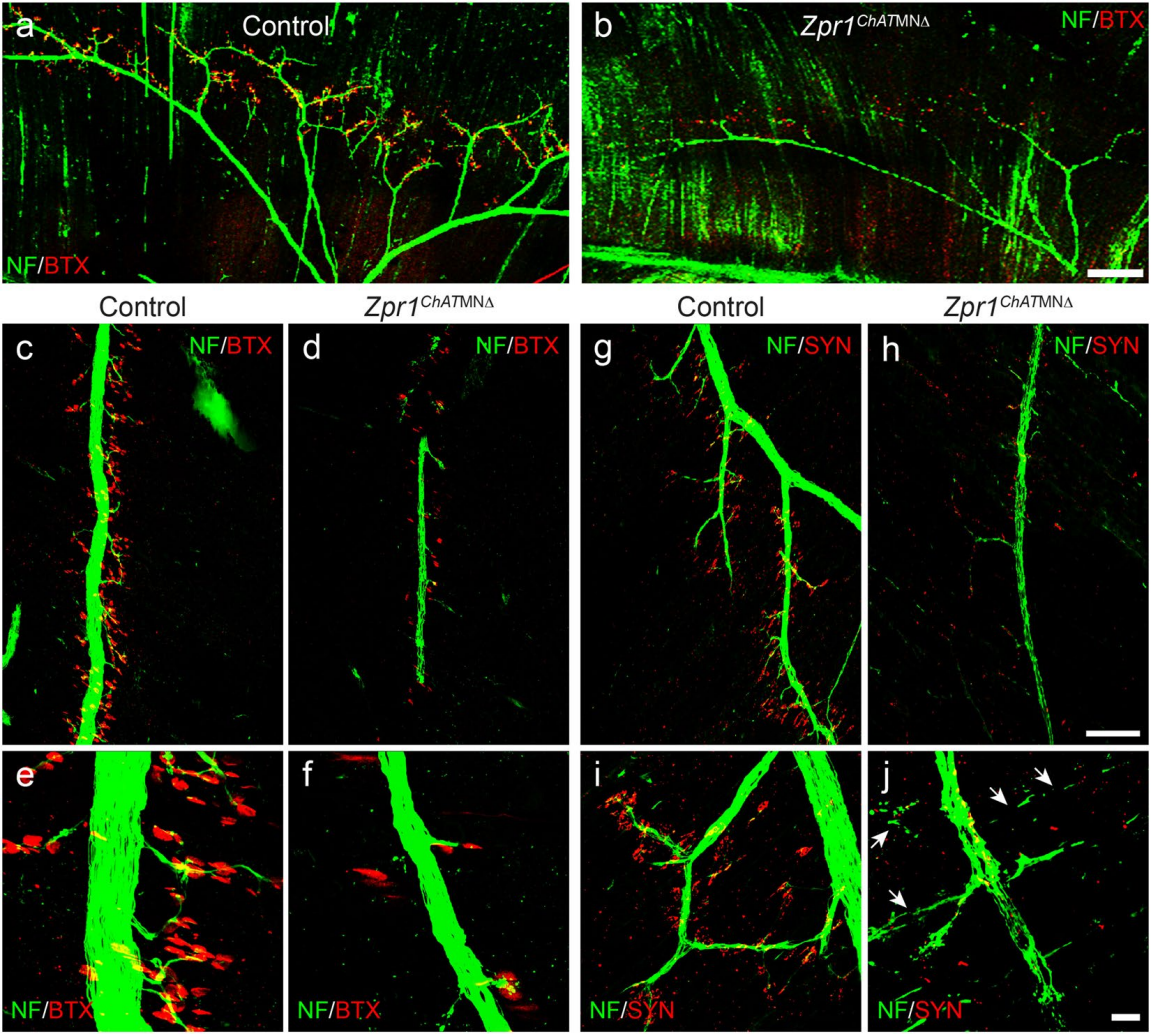
Temporal delay in *Zpr1* mutation causes phrenic nerve degeneration and defects in synapse formation in *Zpr1* ^*ChAT*MNΔ^ mice. (**a**,**b**) Whole diaphragms from control and *Zpr1* ^*ChAT*MNΔ^ E18.5 embryos were stained with neurofilament (NF) antibody and α-bungarotoxin (BTX). Image analysis show defects in branching of the phrenic nerve in *Zpr1* ^*ChAT*MNΔ^ embryos. (**c**–**f**) Analysis of diaphragms stained with NF and BTX shows defects in the formation of NMJs in mutant embryos. Primary innervation of phrenic nerve was improved in *Zpr1* ^*ChAT*MNΔ^ compared to *Zpr1* ^*Hb9*MNΔ^ mice but reduced numbers of secondary and tertiary branches were generated in *Zpr1* ^*ChAT*MNΔ^ mice. (**g**–**j**) Staining of diaphragms with NF and synaptophysin (SYN) shows marked reduction in the tertiary branches and formation of neuromuscular synapses in *Zpr1* ^*ChAT*MNΔ^ mice. Degeneration of secondary and tertiary branches was detected in mutant embryos (arrows). Lack of terminal branches and functional synapses indicate requirement of ZPR1 in motor neurons for the normal functioning of the respiratory system. Scale bars are 250 μm (**a**,**b**), 100 μm (**c**,**d**,**g**,**h**) and 12.5 μm (**e**,**f**,**I**,**j**).

To determine whether PMC neurons in *Zpr1* ^*ChAT*MNΔ^ mice had potential to innervate and arborize in the diaphragm muscle and generate neuromuscular synapses, we examined whole diaphragms from control and *Zpr1* ^*ChAT*MNΔ^ mice, and compared with *Zpr1* ^*Hb9*MNΔ^ mice. Comparison of diaphragms shows that the late removal of *Zpr1* in motor neurons reduces severity of defects in the development of phrenic nerves and improves innervation of diaphragm (Fig. 5). Analysis of diaphragms from E18.5 control and *Zpr1* ^*ChAT*MNΔ^ embryos revealed difference in size and defects in innervation of the phrenic nerve (Fig. 5). Comparison of phrenic nerve diameter between control (21.66 ± 0.014 μm) and *Zpr1* ^*ChAT*MNΔ^ (10.08 ± 0.035 μm) of E18.5 embryos shows (53.28 ± 3.77%, p = 0.000) reduction in diameter and is consistent with the loss of PMC neurons in the spinal cord. However, comparison of phrenic nerve diameter between *Zpr1* ^*Hb9*MNΔ^ and *Zpr1* ^*ChAT*MNΔ^ mice show ~70% increase in the diameter of nerve in *Zpr1* ^*ChAT*MNΔ^ mice that is consistent with the reduced loss of PMC neurons. Diaphragms show improved primary innervation by phrenic nerve in *Zpr1* ^*ChAT*MNΔ^ compared to *Zpr1* ^*Hb9*MNΔ^ mice but reduced secondary and tertiary branches compared to control (Fig. 6a,b). Reduced numbers of innervated NMJs in *Zpr1* ^*ChAT*MNΔ^ compared to control suggest that the motor neurons lacked potential to fully arborize in the diaphragm muscle. Degeneration of secondary and tertiary nerve branches in the diaphragms of *Zpr1* ^*ChAT*MNΔ^ embryos suggests that the neurons with ZPR1 deficiency lacked ability to sustain innervation of NMJs (Fig. 6c–f). Comparison of total number of NMJs between *Zpr1* ^*ChAT*MNΔ^ (12.92 ± 2.04%) and *Zpr1* ^*Hb9*MNΔ^ (9.88 ± 1.34%) mice did not show significant improvement in *Zpr1* ^*ChAT*MNΔ^ mice. However, comparison of innervated NMJs between *Zpr1* ^*ChAT*MNΔ^ (19.44 ± 6.012%) and *Zpr1* ^*Hb9*MNΔ^ (9.560 ± 0.7942%) mice shows small improvement in *Zpr1* ^*ChAT*MNΔ^ mice but not significant (p = 0.154) enough to support restoration of respiration (Supplementary Figure S6). Further, examination of neuromuscular synapse shows normal formation of synapse and co-localization of NF and SYN in neuromuscular junctions in control embryos (Fig. 6g,i). In contrast, diaphragm from mutant *Zpr1* ^*ChAT*MNΔ^ embryos shows severe defects in synapse formation (Fig. 6h,j). Comparison of synapse formation between *Zpr1* ^*ChAT*MNΔ^ and *Zpr1* ^*Hb9*MNΔ^ mice shows improvement in *Zpr1* ^*ChAT*MNΔ^ mice but degeneration of secondary and tertiary nerve branches suggest the loss of functional ability of motor axons to generate synapse. These data suggest that the defects in the formation of synapses may be the cause of respiratory failure and neonatal mortality in *Zpr1* ^*ChAT*MNΔ^ mice. Despite overall reduced severity, including normal anatomical development, reduced loss of PMC neurons and improved innervation of diaphragm by phrenic nerves, all mutant *Zpr1* ^*ChAT*MNΔ^ mice died because of respiratory failure. These data suggest that sustained *Zpr1* gene activity is required for the function of the mammalian respiratory motor neuron system.

### ZPR1 interacts with promoter region of *HoxA5* gene and regulates its expression

**Figure 7:**
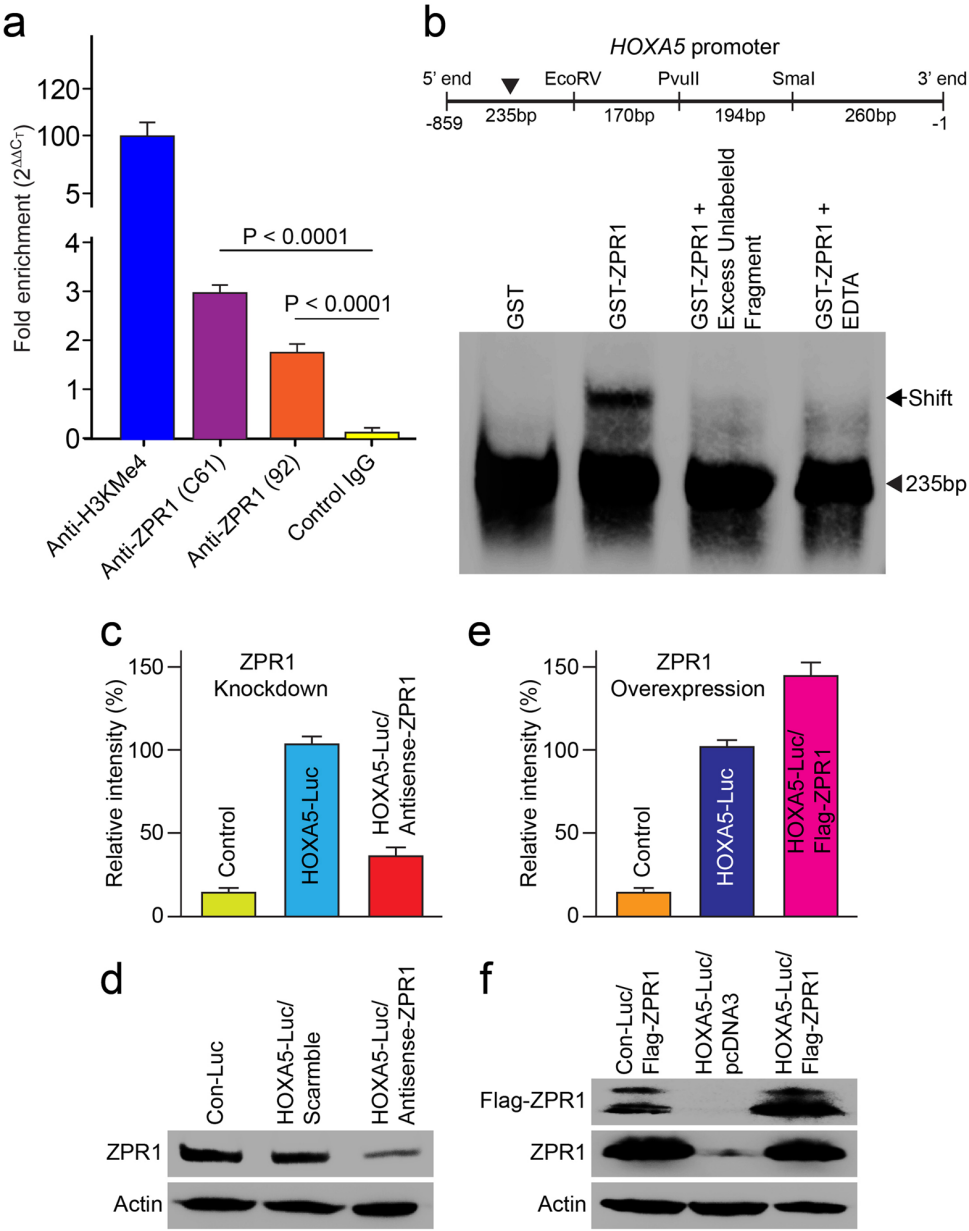
ZPR1 binds to *HoxA5* promoter and regulates expression of the *HoxA5* gene. (**a**) ZPR1 is a part of the chromatin complex. Chromatin samples were prepared using mouse brain tissue. ChIP assay was performed using antibodies against H3KMe4 (positive control) and monoclonal anti-mouseZPR1 (Clone: C61), rabbit polyclonal anti-mouseZPR1 (#92) and anti-Flag M2 (negative control). Real time PCR amplification was performed using universal Taqman PCR reagents and mouse genomic GAPDH primers. Levels of chromatin precipitated with each antibody were quantified using comparative C_T_ (ΔΔC_T_) method for fold enrichment. (**b**) ZPR1 binds human *HoxA5* promoter. EMSA shows that ZPR1 interacts with one of the four fragments (235 bp) tested within ~1 kb region of the human *HoxA5* promoter. (**c**–**f**) Change in levels of ZPR1 influences expression of *HoxA5*-*Luciferase* reporter gene. (**c**,**d**) Effect of ZPR1 knockdown on ectopic *Hox* gene expression in cultured HeLa cells. Cells were transfected with either human HoxA5-Luc or Empty control reporter vector (Con-Luc). Transfected cells were re-transfected after 24 h with scrambled or *Zpr1* antisense oligos to knockdown the levels of ZPR1. Cells were harvested after 24 h post-second transfection for determination of luciferase activity or immunoblot analysis. Quantification of luciferase activity (mean ± s.e.m.; n = 3) shows ZPR1 knockdown reduced levels of luciferase activity to 32.77 ± 7.91%, p = 0.000 in cells treated with antisense oligos. (**e**,**f**) To examine the effect of ZPR1 overexpression, HeLa cells were transfected with combination of two plasmids (i) Con-Luc + pcDNA3-*FlagZPR1*, (ii) HoxA5-Luc + pcDNA3 (empty) and (iii) HoxA5-Luc + pcDNA3*FlagZPR1*. After 30 h post-transfection, cells were harvested for determining luciferase activity or immunoblot analysis. Quantification of luciferase activity shows ZPR1 overexpression results in increase of luciferase activity (42.80 ± 13.72%, p = 0.011) compared to cells without ZPR1 overexpression (Full-length blots are included in Supplementary Figure S7).

The loss of HoxA5^+^ neurons and reduced staining of HoxA5 in the spinal cords in *Zpr1* mutant mice (Fig. 3a,e) suggest ZPR1-defciency might be a cause of deregulation of HoxA5 levels because sustained levels of HoxA5 are required for the survival and function of phrenic motor neurons^17^. It is possible that ZPR1 might be a regulator of *HoxA5* expression because ZPR1 is a C4-type zinc finger protein and has been shown to be involved in the regulation of transcription^29^. To test whether ZPR1 acts as a transcription factor and regulates the expression of *HoxA5* gene, we examined (a) interaction of ZPR1 with *HoxA5* promoter region and (b) the effect of change in ZPR1 levels on the expression of *HoxA5* gene. Immunoprecipitation of chromatin using antibodies against ZPR1 shows interaction of ZPR1 with genomic DNA (Fig. 7a). Electrophoretic mobility-shift assay shows that ZPR1 interacts with one of the four fragments (235 bp) tested within ~1 kb region of the human *HoxA5* promoter (Fig. 7b). To test the effect of ZPR1 on the transcription, we utilized *in vitro* approach and used a plasmid containing *Luciferase* gene under the control of human *HoxA5* promoter and transfected HeLa cells to measure the luciferase enzyme activity. The levels of ZPR1 were (i) down-regulated by knockdown with antisense oligonucleotide^29^ and (ii) up-regulated by transfection of plasmid containing recombinant human *Flag*-*ZPR1* cDNA^14^. Knockdown of ZPR1 levels in cells expressing *HoxA5*-*Luciferase* results in reduced luciferase activity (32.77 ± 7.91%, p = 0.000) compared to cells treated with scrambled oligonucleotide (Fig. 7c,d). The decrease (~67%) in *HoxA5* promoter activity is consistent with *in vivo* reduction in the levels of HoxA5 protein caused by *Zpr1* gene mutation in *Zpr1* ^*Hb9*MNΔ^ and *Zpr1* ^*ChAT*MNΔ^ mice. Importantly, overexpression of ZPR1 results in increase of luciferase activity (42.80 ± 13.72%, p = 0.011) compared to cells without ZPR1 overexpression (Fig. 7e,f). These data show that the modulation of ZPR1 levels results in alteration of *HoxA5* expression in direct correlation with ZPR1 levels. These findings suggest that modulation in ZPR1 levels directly correlates and influences levels of *HoxA5* transcription and ZPR1 may be a transcriptional regulator of the *HoxA5* gene.

### ZPR1 deficiency causes down regulation of SMN and HoxA5 in motor neurons

**Figure 8:**
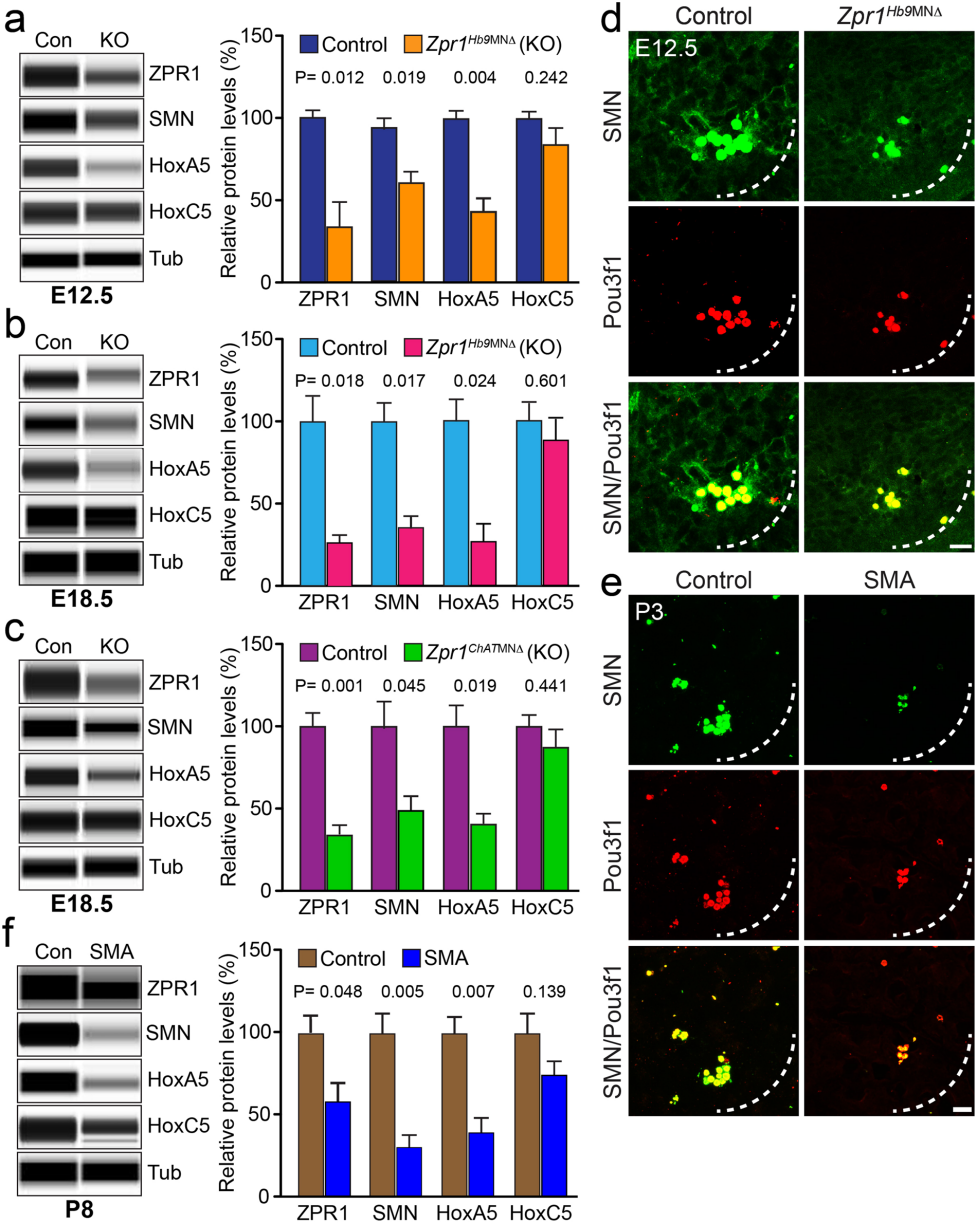
Deregulation of ZPR1 results in down regulation of HoxA5 that causes respiratory distress in *Zpr1* mutant and SMA mice. Protein levels of ZPR1, SMN, HoxA5 and HoxC5 were examined in the spinal cords from control and mutant E12.5 and E18.5 embryos using automated Protein Wes System (ProteinSimple) and quantification was performed using Compass software. Quantitative analysis (mean ± s.e.m., n = 3 mice/group) of protein levels show that *Zpr1* mutation resulted in reduced protein levels in (**a**) E12.5 embryos ZPR1 (47.44 ± 11.98%, p = 0.012), SMN (73.13 ± 5.14%, p = 0.019), HoxA5 (43.62 ± 8.84%, p = 0.004) and HoxC5 (87.23 ± 8.81%, p = 0.242) and (**b**) E18.5 embryos ZPR1 (27.67 ± 4.97%, p = 0.010), SMN (35.33 ± 7.86%, p = 0.011), HoxA5 (29.00 ± 10.44%, p = 0.024) and HoxC5 (89.00 ± 14.22%, p = 0.637) of *Zpr1* ^*Hb9*MNΔ^ mice compared to control mice. (**c**) Protein levels in *Zpr1* ^*ChAT*MNΔ^ mice also shows reduced levels for ZPR1 (33.67 ± 3.75%, p = 0.001), SMN (50.33 ± 8.21%, p = 0.045), HoxA5 (42.33 ± 5.84%, p = 0.019) and HoxC5 (88.00 ± 12.17%, p = 0.441) compared to control at E18.5 stage. (**d**) Spinal cord sections of the cervical region (C3-C5) from E12.5 control and *Zpr1* ^*Hb9*MNΔ^ embryos were stained with Pou3f1 and SMN antibodies. Immunofluorescence analysis shows ZPR1-deficiency causes loss of SMN^+^ and Pou3f1^+^ phrenic motor neurons in *Zpr1* ^*Hb9*MNΔ^ mice. (**e**) Cervical region spinal cord sections from 3-days old (P3) severe SMA mice show that SMN-deficiency causes loss of SMN^+^ and Pou3f1^+^ phrenic motor neurons similar to ZPR1-deficiency in *Zpr1* ^*Hb9*MNΔ^ mice. Scale bar is 10 μm. (f) Quantification of protein levels in the spinal cords from P8 SMAΔ7 mice show that SMN deficiency (69.10 ± 12.42%, p = 0.005) reduces protein levels to: ZPR1 (58.87 ± 10.43%, p = 0.048), HoxA5 (39.41 ± 9.03%, p = 0.007) and HoxC5 (74.90 ± 7.06%, p = 0.139) proteins in SMAΔ7 mice compared to control mice (Full-length blots are included in Supplementary Figures S8 and S9).

To unravel the molecular mechanism of respiratory failure in mutant *Zpr1* ^*Hb9*MNΔ^ and *Zpr1* ^*ChAT*MNΔ^ mice caused by ZPR1 deficiency and its correlation with respiratory distress in SMA pathogenesis, we examined protein levels of SMN, HoxA5 and HoxC5 in the spinal cords from E12.5 and E18.5 control and mutant embryos. Quantitative analysis of protein levels in E12.5 spinal cords shows that the *Zpr1* gene mutation reduced levels of proteins to, ZPR1 (47.44 ± 11.98%, p = 0.012), SMN (73.13 ± 5.14%, p = 0.019), HoxA5 (43.62 ± 8.84%, p = 0.004) and HoxC5 (87.23 ± 8.81%, p = 0.242) in *Zpr1* ^*Hb9*MNΔ^ mice compared to control mice (Fig. 8a). Protein levels were further reduced to ZPR1 (27.67 ± 4.97%, p = 0.010), SMN (35.33 ± 7.86%, p = 0.011), HoxA5 (29.00 ± 10.44%, p = 0.024) and HoxC5 (89.00 ± 14.22%, p = 0.637) in E18.5 *Zpr1* ^*Hb9*MNΔ^ mice compared to control mice (Fig. 8b). Similarly, estimation of protein levels in *Zpr1* ^*ChAT*MNΔ^ mice also shows reduced levels for ZPR1 (33.67 ± 3.75%, p = 0.001), SMN (50.33 ± 8.21%, p = 0.045), HoxA5 (42.33 ± 5.84%, p = 0.019) and HoxC5 (88.00 ± 12.17%, p = 0.441) compared to control at E18.5 stage (Fig. 8c). These data suggest that *Zpr1* mutation causes significant reduction in the levels of SMN and HoxA5 but not in the levels of HoxC5 that shows the specificity of down-regulation HoxA5 in *Zpr1* mutant mice. Reduction in levels of SMN ~65% and HoxA5 ~71% (*Zpr1* ^*Hb9*MNΔ^) and SMN ~50% and HoxA5 ~52% (*Zpr1* ^*ChAT*MNΔ^) in mutant mice correlate with the greater severity of phenotype in *Zpr1* ^*Hb9*MNΔ^ compared to *Zpr1* ^*ChAT*MNΔ^ mice. It is established that reduction in SMN protein levels by ~70%, ~50% and ~30% correlates with severity of SMA disease in type I, II and III patients, respectively^30, 31^. Therefore, reduction in SMN protein levels by ~65% (*Zpr1* ^*Hb9*MNΔ^) and ~50% (*Zpr1* ^*ChAT*MNΔ^) suggest development of severe to moderate SMA-like disease in *Zpr1* mutant mice.

### SMN deficiency causes down regulation of ZPR1 and HoxA5 and degeneration of phrenic motor neurons in SMA mice

To test whether SMN deficiency causes degeneration of phrenic motor neurons and contributes to the respiratory distress associated with SMA pathogenesis, we examined cervical region of the spinal cord from mice with severe SMA that has mean survival of 4–5 days. We exploited the opportunity to examine phrenic motor neurons in postnatal (P3) mice because it was not possible to examine *Zpr1* mutant mice due perinatal lethality. SMN deficiency causes the loss of SMN^+^ phrenic motor neurons in SMA mice similar to ZPR1 deficiency (Fig. 8d,e) suggesting that ZPR1 and SMN deficiency cause similar degeneration of phrenic motor neurons that may contribute to respiratory distress in SMA. To identify common molecular targets involved in phrenic motor neuron degeneration caused by ZPR1 and SMN deficiencies that may contribute to respiratory distress in SMA, we examined the levels of ZPR1, HoxA5 and HoxC5 in a less severe SMA mouse model SMAΔ7. Quantitative analysis of protein levels in spinal cords from postnatal (P8) mice show that SMN deficiency (69.10 ± 12.42%, p = 0.005) reduces levels of ZPR1 (58.87 ± 10.43%, p = 0.048), HoxA5 (39.41 ± 9.03%, p = 0.007) and HoxC5 (74.90 ± 7.06%, p = 0.139) proteins in SMAΔ7 mice compared to control mice (Fig. 8f). Small and non-significant change in HoxC5 is consistent with data from *Zpr1* mutant mice and serves as a control to support the specificity of defect caused by either ZPR1 or SMN deficiency. Comparison of data from mutant *Zpr1* ^*Hb9*MNΔ^ and *Zpr1* ^*ChAT*MNΔ^ mice with SMAΔ7 mice show that ZPR1 deficiency results in deregulation SMN and HoxA5 levels and SMN deficiency results in down-regulation of ZPR1 and HoxA5. Together, these findings suggest that the low levels of SMN, ZPR1 and HoxA5 collectively contribute to the respiratory distress associated with SMA pathogenesis.

## Discussion

Breath (*Prana* in Sanskrit or *Spiritus* in Latin) or the act of breathing, respiration, is essential for all vertebrates living on the planet Earth. Respiratory failure is the cause of death in SMA^3^. Molecular mechanisms underlying respiratory distress in SMA are unknown. Respiration rhythm in mammals is maintained by motor activity of diaphragmatic muscle controlled by motor neurons of the phrenic nerve, which originates in the cervical region (C3-C5) of the spinal cord and is the only source of motor neuron supply to the diaphragm^32, 33^. The *Hox* genes are known to control the differentiation, identity and columnar organization of specific groups of motor neurons in the spinal cord^34^. Genetic studies on mutation of the *Hox5* group of genes (*Hoxa5*, *Hoxb5* and *Hoxc5*) in mice have shown that *Hoxa5* is critical for the development and function of the phrenic nerve motor neurons^17, 22, 23^. In this study, we found that ZPR1 is critical for the function of phrenic motor neurons that regulate respiration. ZPR1 functions downstream of SMN and transcriptionally regulates HoxA5 levels in motor neurons. In SMA, SMN-deficiency causes deregulation of ZPR1 and HoxA5 that lead to respiratory failure.

### ZPR1 is an evolutionary conserved signaling protein

The *Zpr1* gene (*Chr 11q23*.*2* in humans) is evolutionary conserved in eukaryotes, yeast to mammals, and encodes protein with unique modular architecture consisting of two C4-type Zn^2+^ fingers (ZnF1 and ZnF2) with a common topology and two domains termed A and B, which are homologous to each other but not in other proteins. These domains occur in a tandem order (ZnF1-A domain-ZnF2-B domain) that is broadly conserved in eukaryotic organisms^6^. Genetic studies have shown that the core function of ZPR1 is preserved during evolution and the *Zpr1* gene is essential for viability in yeast and mice^5, 8, 35^. However, the biological function of ZPR1 is unknown. Biochemical studies have provided insight that ZPR1 domains interact with distinct proteins: A-domain interacts with eEF1A and the B-domain interacts with SMN protein^5, 9^. In addition, both Zinc-Fingers interact with cytoplasmic domain of EGFR and PDGFR that is highly conserved among RTKs^4^. In mammalian cells, ZPR1 binds to inactive EGFR and PDGFR and upon receptor activation by EGF or serum, ZPR1 translocate to the nucleus^4, 35^. In the nucleus, ZPR1 accumulates in sub-nuclear bodies, SMN^+^ gems, histone-locus body and CB^9, 29^. Genetic mutation study in *Drosophila* has shown that the ZPR1 mediates effects of EGFR and fibroblast growth factor receptor (FGFR) signaling required for lumen formation in terminal cells^7^. Together these findings, including evolutionary conservation and ubiquitous expression suggest that ZPR1 may be a common downstream target of multiple RTKs, including EGFR, PDGFR and FGFR that mediates growth signaling in cells and tissues throughout the lifespan.

### ZPR1 is critical for the development and function of respiratory motor neurons

*Zpr1* mutation resulted in defects in the development and function of the respiratory system in *Zpr1* ^*Hb9*MNΔ^ and *Zpr1* ^*ChAT*MNΔ^ mice. These defects included under developed lungs, loss of PMC neurons, inability of motor neurons to innervate diaphragm muscle and populate NMJs and lack of neuromuscular synapse. Remarkable extent of severity was displayed by complete absence of phrenic nerve or partial presence of nerve only on one side of the diaphragm in *Zpr1* ^*Hb9*MNΔ^ mice suggesting that the development and targeting of phrenic nerve neurons to the diaphragm was impaired by ZPR1 deficiency. Respiratory failure and perinatal lethality have been reported in mice with mutation of the *Hb9* ^−/−^ gene^19^ and *Hox5* ^MNΔ^ (*Hoxa5* ^*loxP*/*loxP*^; *Hoxc5* ^−/−^; *Olig2*::*Cre* mice)^17^. Defects in lungs morphogenesis observed in *Zpr1* ^*Hb9*MNΔ^ mice have been reported in other mutant mice, *Hb9* ^−/−^, *Hoxa5* ^−/−^, *Hox5* ^MNΔ^, (*Hoxa5* ^−/−^; *Hoxb5* ^−/−^)^17, 19, 23, 36^, however, the severity of defects is most pronounced in *Zpr1* ^*Hb9*MNΔ^ mice compared to other mutant mice. Similarity of defects in lungs morphogenesis in mice with conventional knockouts and conditional knockouts in motor neurons suggest that the upper spinal cord neurons may be involved in the regulation of lungs morphogenesis during embryogenesis^16, 37^. However, which specific region and group of neurons controls lungs development remains to be identified and warrants further studies? It is possible that the paralogs of *Hox* genes and *Zpr1* may be components of a common mechanism that control development of mammalian respiratory system, including lungs morphogenesis.

Temporal delay in *Zpr1* mutation preserved anatomical and skeleton development in *Zpr1* ^*ChAT*MNΔ^ mice. Although the severity of defects in the innervation of diaphragm was reduced but the perinatal lethality of mice could not be rescued and mice died immediately after birth because of respiratory failure. Temporal analysis of ZPR1 function and comparison of phenotypes of *Zpr1* ^*Hb9*MNΔ^ and *Zpr1* ^*ChAT*MNΔ^ mice revealed that phrenic motor neurons are most sensitive to the low levels of ZPR1 and sustained *Zpr1* gene activity is required for the function of respiratory motor neurons.

### Molecular mechanism of respiratory failure in SMA

Severe SMA Type I (Werdnig-Hoffman syndrome) patients require ventilation for life support after birth and live ~2 years. Primary SMA pathogenesis shows degeneration of motor neurons in the lumbar region that results in muscle atrophy^3^. How SMN-deficiency leads to respiratory failure in SMA is unclear? Findings of this study provide insight into the molecular events stemming downstream of SMN-deficiency that lead to degeneration of phrenic nerve motor neuron and respiratory failure in SMA.

 Defects in intracellular signaling downstream of growth factor receptors because of low levels of SMN and ZPR1 might account for degeneration of phrenic motor neurons and respiratory distress in SMA. Previous studies have shown that ZPR1 binds to the highly conserved cytoplasmic domain of inactive growth receptors (EGFR, PDGFR) and the treatment of quiescent cells with serum containing growth factors results in the formation ZPR1-SMN protein complex and translocation of ZPR1-SMN complex from the cytoplasm to the nucleus in mammalian cells^4, 9^. *Drosophila* FGF receptor (*breathless*, *btl*) was identified as a modifier of *Smn*-dependent lethality by a genetic screen, suggesting a link between SMN and FGF signaling pathway^38^. Further, activation of FGF signaling in muscle rescues synaptic defects in neuromuscular junctions caused by SMN deficiency^39^. Intracellular FGF-2 isoform interacts with SMN and regulates stability of SMN containing sub-nuclear bodies^40^. ZPR1 is shown to be a downstream component of EGFR and FGFR signaling pathways and mediates development of trachea (respiratory system) in *Drosophila* ^7^. Interestingly, down regulation of SMN levels in *Drosophila* also resulted in a phenotype similar to ZPR1 deficiency, suggesting both ZPR1 and SMN are components of a common pathway that regulates development of the respiratory system^7^.

The results of this study demonstrate that the low levels of ZPR1 cause down-regulation of HoxA5 that leads to degeneration of phrenic motor neurons and respiratory failure in mice. SMA patients express reduced levels of ZPR1^14, 15^, our data indicate that low levels of ZPR1 may contribute to respiratory distress in SMA. In SMA mice, low levels of SMN cause deregulation of ZPR1 and HoxA5 that result in degeneration of phrenic motor neurons leading to respiratory distress and death. Respiratory distress caused by the low levels of HoxA5 in *Zpr1* mutant mice and SMA mice is consistent with finding that HoxA5 deficiency causes degeneration of phrenic motor neurons and respiratory failure in mice^17^. The FGF/FGFR pathway has been shown to regulate *Hox* gene expression during development^41, 42^. Deregulation of HoxA5 in motor neurons of two *Zpr1* mutant (*Zpr1* ^*Hb9*MNΔ^ and *Zpr1* ^*ChAT*MNΔ^) mice and SMA mice suggest that the normal levels of ZPR1-SMN complex are required to maintain optimal levels of HoxA5 in motor neurons. Because ZPR1 binds to inactive RTKs and translocate from the cytoplasm to the nucleus in response to receptor activation, it is possible that ZPR1 acts downstream of FGFR signaling and form complex with SMN to regulate expression of *Hoxa5* gene. Recently, SMN-Pol II complex has been shown to be involved in regulation of transcription termination^43^. We show that increase in ZPR1 levels enhances transcription of *HoxA5* and because ZPR1-SMN complex is localized in the nucleus it is possible that ZPR1 and SMN proteins are parts of transcription complex that regulates expression of *HoxA5*. Further, zinc fingers of ZPR1 have similarity to nucleic acid interacting proteins^6^. A recent study showed that plant (*Solanum tuberosum*) StZPR1 binds to a DNA motif ‘CAACAGCATC’, present in the promoter of a clock-controlled double B-box *StBBX24* gene and regulate its expression, and suggested that ZPR1 may be a transcription regulator^44^. However, this class of plants specific light-regulated circadian clock genes is absent in mammals. Similarly, plants lack *Hox* genes. Our initial analysis showed that ZPR1 interacts with a 235 bp DNA fragment of human *HoxA5* promoter. Further, analysis identified a 20 bp DNA sequence ‘CTGCGGGCAGGATTTATTTCTCCAATT’ that contains Oct3/4 binding motif ‘AGGATTTATTT’. Interestingly, *in silico* analysis shows that a 17 bp DNA sequence ‘GGGCAGGATTTATTTCT’ containing Oct3/4 binding motif is present in the both mouse and human *HoxA5* promoters but not in the *HoxC5* promoters suggesting that ZPR1 might regulate expression of *HoxA5* but not of *HoxC5*. This is consistent with our data from mutant *Zpr1* ^*Hb9*MNΔ^ and *Zpr1* ^*ChAT*MNΔ^ mice that show ZPR1 deficiency causes significant decrease in levels of HoxA5 but not of HoxC5. SMN-deficiency causes deregulation of ZPR1 that result in down-regulation of HoxA5 but did not reduce levels of HoxC5 significantly suggesting combined deficiency of SMN and ZPR1 selectively decreases levels of HoxA5 in phrenic motor neurons. In conclusion, ZPR1 is a critical regulatory factor that may functions in collaboration or downstream of SMN to regulate levels of HoxA5 in phrenic motor neurons that control respiration. Together, these findings provide insight into the molecular basis of respiratory failure associated with SMA pathogenesis and identify ZPR1 and HoxA5 as potential targets that lay a foundation for developing therapeutic strategies to treat respiratory distress in SMA.

## Methods

All experiments and procedures were approved and performed according to the guidelines and policies set by the Institutional Biosafety Committee (IBC). All animals were housed in a facility accredited by the Association for Assessment and Accreditation of Laboratory Animal Care (AAALAC). All animal experiments were approved by the Institutional Animal Care and Use Committee (IACUC) of the Texas Tech University Health Sciences Center El Paso (TTUHSC EP). Animals were treated humanly and euthanasia was performed using methods approved by the American Veterinary Medical Association (AVMA).

### Creation of mice with a conditional allele of the *Zpr1* gene

The murine *Zpr1* gene was isolated from a 129/SvJ mouse λ Fix II genomic library (Stratagene) using the mouse *Zpr1* cDNA as a probe. Sequence analysis confirmed that the clone contains the *Zpr1* gene^8^. A targeting vector was designed to replace *Zpr1* exon 1 with *loxP*-flanked exon 1 and a *loxP*-flanked *Neo* ^*R*^ (floxed) cassette (Figure S1a). A thymidine kinase (TK) cassette was included for negative selection. The targeting vector was linearized with *Not*I and electroporated into 129SvEvTac (Taconic) embryonic stem cells and subsequently selected with G418 and gancyclovir (Ingenious Targeting Laboratory, New York). Targeted clones were identified by Southern blot analysis using a random-primed ^32^P-labeled 425 bp probe prepared by PCR using primer pair CTCAGACATGACCAGAGACC and CCGCTGTGGGGCCAGGCCCG and mouse *Zpr1* cDNA as the template (Figure S1b). Targeted ES cells were used to create chimeric mice by blastocyst injection. Germ-line transmission of floxed exon 1 *Zpr1* allele with floxed *Neo* ^*R*^ cassette was achieved by crossing chimeric mice to C57BL/6J strain (The Jackson Laboratory). The *Neo* ^*R*^ cassette was removed *in vivo* by crossing mice with disrupted *Zpr1* allele containing floxed *Neo* cassette with Protamine-Cre (*PC3*-*Cre*) mice that expresses Cre recombinase in male germ-line^45^ to generate mice with a conditional allele containing floxed exon 1 (*Zpr1* ^+/F1^) (Supplementary Figure S1a). Genomic DNA was isolated from the mouse-tail with DNA isolation kit (Lamda Biotech) and used for genotyping by PCR method using primers; Forward (EX1-F4: CCCTCAGCGCCGAGGATGAG) and Reverse (EX2-R4: GCAGGAAAAGGAGCTCACGA) that generate 249 bp fragment (wild-type allele) and 289 bp fragment (floxed allele) (Supplementary Fig. S1c). Heterozygous mice *Zpr1* ^+/F1^ were crossbred to generate mice with homozygous conditional allele *Zpr1* ^F1/F1^. Transgenic mice *Hb9*-*Cre* (B6;129S1-*Mnx1* ^tm4(cre)Tmj^/J, Stock No: 006600) and *ChAT*-*Cre* (B6;129S1-*ChAT* ^tm2(cre)Lowl^/J, Stock No: 006410) were purchased from the Jackson Laboratory. The other mouse models used in this study were, severe SMA [*Smn* ^−/+^; *SMN2* ^+/+^] and SMAΔ7 [*Smn* ^−/+^; *SMN2* ^+/+^; *SMN*Δ*7* ^+/+^]^46^, purchased from the Jackson Laboratory.

### Histology

Mouse tissues, lungs, diaphragm and spinal cords from control and mutant E18.5 embryos were isolated, fixed in 4% paraformaldehyde (PFA) at 4 °C for overnight, washed with PBS and then soaked in 30% sucrose solution for 48 h. Tissues were processed and embedded in OCT for frozen sections. Thin serial sections (10 µm) were cut from the cervical region (C2-C6), thoracic region (T9-T12) and lumbar region (L1-L5) of the spinal cords and stained with hematoxylin and eosin or processed for immunohistochemical staining. Motor neurons were counted in every fifth section (20 sections) of the regions of the spinal cord^14, 18^. For immunohistochemical staining, sections were processed for antigen retrieval, briefly incubated in SDS (1%) solution for 5 min, and another 5–10 min in 0.1 M sodium citrate buffer maintained at 95 °C. Sections were rinsed and incubated with primary antibodies against ZPR1 (Clone # LG-D5), ChAT (#ab6168, Abcam), diluted 1:100 with antibody diluent from the M.O.M kit (Vector Laboratories). Immune complexes were detected by using a biotinylated secondary antibody, streptavidin-conjugated horseradish peroxidase and the substrate 3,3′-diaminobenzene followed by brief counterstaining with hematoxylin.

### Immunofluorescence analysis

Control or mutant E12.5 and E18.5 days-old embryos were collected and fixed overnight in 4% PFA. Next day fixed embryos were washed with PBS for 4 h and spinal cord, fore and hind limbs, whole diaphragm were carefully isolated and washed with PBS and soaked in (30%) sucrose solution for 48 h. Tissues were embedded in OCT for frozen sections. Thin serial spinal cord (10 μm) and hind limb (30 μm) sections were blocked in 3% BSA with 0.1% Triton-X100 for an hour and then incubated overnight with primary antibodies against HoxA5 and HoxC5 (Dr. T. Jessell, Columbia University)^28^, Pou3f1 (Dr. D. Meijer, University of Edinburgh)^47^, Islet1/2 (Developmental Study Hybridoma Bank, DSHB, #39.4D5), FoxP1 (#ab16645, Abcam), ZPR1 (Clone LG-D5) diluted (1:100) in 3% BSA. Sections were washed 5 times in PBST, incubated for an hour with Alexa 488-conjugated anti-mouse or rabbit IgG secondary antibody diluted to 1:400 in 3%BSA, washed 4X with PBST, and mounted with DAPI containing Vectashield (Vector Lab). Whole diaphragm was blocked in Mouse-blocking solution (MOM kit; Vector Labs) containing 0.3% Triton-X100 for 4 h. Diaphragms were incubated in neurofilament primary antibody (NF-M, 145 kDa) protein (#MAB1621, Millipore) for 24 h followed by Alexa488-conjugated anti-mouse secondary antibody after washing them in PBS (5 × 10 min). For double staining the diaphragms were again incubated in Mouse-blocking solution for 30 min, rinsed with PBS (3 × 5 min) and then incubated with α-Bungarotoxin (1:100, Abcam) conjugated with Alexa596 for 2 h and washed 5x with PBS before mounting on glass slides using fluorescence mounting medium (Vector Labs) and cover-slipped. For double staining of diaphragms with NF and synaptophysin, the protocol for blocking and NF staining was similar as mentioned above. Synaptophysin (1:100, ab14692, Abcam) staining was done overnight, tissues washed with PBS (5 × 10 min), incubated with anti-rabbit Alexa596 conjugated secondary antibody for 4 h and washed 5 times with PBS before mounting on microscope slides using florescence mounting medium (Vector Labs) and cover-slipped^48^. Whole diaphragm images were captured with 10x objective using TileScan mode and were auto-stitched and merged using Leica Software. For z-stack and other images, the z-volume and other acquisition parameters were constant for each experiment. Images were captured using confocal microscope (Leica-TCS-SP5, Leica Microsystems) equipped with AOBS and UV (405 nm), IR (633 nm) and Visible range lasers.

### Immunoblot Analysis

Spinal cords were isolated from control and mutant E18.5 embryos. Tissue lysates were prepared and diluted to protein concentration of 0.4 μg/μl using sample preparation kit (ProteinSimple). Tissue lysates were examined using automated western blot system, WES System (ProteinSimple) as per manufacturer’s protocol. The following primary antibodies were used for analysis: ZPR1 (Clone LG-C61), Flag M2 (#F1804, Sigma), HoxA5, HoxC5, α-tubulin (#T8203, Sigma) and β-Actin (#A5316, Sigma). Data analysis and quantitation of protein levels were performed using Compass Software (Protein Simple). For standard immunoblot method, cell lysates were prepared from control and transfected HeLa cells using Triton lysis buffer^5^. Proteins were separated by SDS-PAGE and electrotransferred to PVDF membrane (Millipore). Proteins were detected using primary antibodies (1:100), ZPR1 (Clone LG-C61), Flag M2 and α-tubulin (#T6074, Sigma) followed by HRP-conjugated donkey anti-mouse or rabbit IgG (1:5000) secondary antibodies. Chemiluminescence and quantitation of immunoblots was performed using ImageQuant LAS4000. The relative levels of proteins (mean ± s.e.m.) normalized to either actin or tubulin, are presented as bar graphs.

### ChIP-Real-time PCR Assay

Chromatin samples were prepared using mouse brain tissue. ChIP assay was performed using antibodies against H3KMe4 as a positive control (#NB21–1023, Novas Biologicals) and monoclonal anti-mouseZPR1 (Clone: LG-C61), rabbit polyclonal anti-mouseZPR1 (#92) and anti-Flag M2 (negative control) according to manufacturer's protocol of the ﻿Magna ChIP™ HiSens kit (Millipore, 17–10460). Real-time PCR amplification was performed using universal Taqman PCR reagents and inventoried mouse genomic GAPDH primers (AB-4167A, Life Technologies). Levels of chromatin precipitated with each antibody were quantified using comparative C_T_ (ΔΔC_T_) method for fold enrichment.

### Electrophoretic Mobility-Shift Assay

A 235 bp DNA fragment from human *HoxA5* promoter was amplified by PCR using primers, Forward: 5′-ATATTCACACGAAAGAAAAATCG-3′ and Reverse: 5′-ATCACCTTCGGGG AAGG-3′. A small 20 bp oligonucleotide (CTGCGGGCAGGATTTATTTCTCCAATT) contains Oct3/4 binding motif from the 235 bp sequence was synthesized (IDT). DNA fragments were end-labeled using Biotin 3′-end DNA labeling kit (Thermofisher Scientific). Recombinant GST and GST-ZPR1 proteins were produced in bacteria (BL21) using PGEX-5x-2 (GST) and PGEX-5x-2/*GST*-*humanZPR1* vectors and purified using GST-Agarose beads column^5^. Electrophoretic Mobility-Shift (EMSA) assay was performed using labeled DNA probe and GST and GST-ZPR1 proteins using light shift chemiluminescent EMSA kit according to the manufacturer’s instruction (Thermofisher Scientific). Briefly, biotin labeled *HoxA5* DNA probe was added to 10X binding buffer, 100 mM MgCl_2_, 1% NP 40, 50% Glycerol, 1 ug/uL poly (dI.dC) and purified GST or GST-ZPR1 protein. Excess (100-folds) unlabeled probe was used for competition and 20 mM EDTA (final concentration) was used for chelating Zn^2+^ to collapse zinc fingers and disrupt protein-DNA interaction. The reaction mixture was incubated for 30 min and complexes were separated on a 5% native polyacrylamide gel by electrophoresis, electro-transferred to a nylon membrane at 100 V for 1 h. Transferred DNA-protein complexes were crosslinked by UV (120 mJ/cm^2^ for 60 sec) using a UV-light crosslinker (Spectrolinker XL-1500, Spectronics Corp.). Biotin-labeled DNA probes were detected using the streptavidin-HRP conjugate and chemilumeniscence.

### Ectopic *Hox* Gene Expression Analysis

To examine the effect of change in the levels of ZPR1 on alteration of the *Hox* gene expression, we used HeLa cells co-transfected with human *HoxA5* promoter driven luciferase reporter vector (HoxA5-Luc) (Affymetrix) and either pcDNA3-*FlagZPR1* (ZPR1 overexpression)^14^ or with *Zpr1*-antisense oligonucleotides (ZPR1 knockdown)^29^. To examine the effect of ZPR1 knockdown, cultured HeLa cells were transfected with either human HoxA5-Luc or Empty control reporter vector (Con-Luc) using lipofectamine reagent (Invitrogen). Transfected cells were re-transfected after 24 h with scrambled or *Zpr1* antisense oligos to knockdown the levels of ZPR1. Cells were harvested after 30 h post-second transfection for determination of luciferase activity or immunoblot analysis. To examine the effect of ZPR1 overexpression, HeLa cells were transfected with combination of two plasmids (i) Con-Luc + pcDNA3-*FlagZPR1*, (ii) HoxA5-Luc + pcDNA3 (empty) and (iii) HoxA5-Luc + pcDNA3*FlagZPR1* using the lipofectamine reagent. After 30 h post-transfection, cells were harvested for determining luciferase activity or immunoblot analysis. Luciferase activity was measured using the Luciferase assay system (Promega) in the synergy H1 hybrid multi-mode microplate reader (BioTek Instruments). Relative levels of luciferase activity (mean ± s.e.m.) are presented as bar graphs.

### Statistical analysis

The quantitative data is presented as mean ± s.e.m. Statistical analysis performed using Student’s t-test (two tailed) with GraphPad Prism (version 5.0d). The value p = 0.05 or less was considered significant. In all experiments with mice, ‘*n*’ represents the number of embryos of﻿ mice used per group and in experiments with tissues or cells ‘*n*’ represents the number of times experiment was performed. A minimum of n = 3 embryos of mice per genotype or number of times experiment performed was used in all the experiments, unless otherwise specified in an experiment.

### Data availability

All data generated and analysed during this study are included in this published article (and its Supplementary Information files).

## Electronic supplementary material

Supplementary Information

Movie S1
