# Supplementary material for: Deregulation of ZPR1 causes respiratory failure in spinal muscular atrophy
Source: Sci Rep. 2017 Aug 15;7:8295. doi: 10.1038/s41598-017-07603-z (PMC5557895; doi:10.1038/s41598-017-07603-z)
Supplement: Supplementary file 1 — Supplementary Information [file 41598_2017_7603_MOESM1_ESM.pdf]

SUPPLEMENTARY INFORMATION

Deregulation of ZPR1 causes respiratory failure in spinal muscular atrophy

Naresh K. Genabai[1,2], Annapoorna Kannan[1,2], Saif Ahmad[1,2], Xiaoting Jiang[1,2,3], Kanchan Bhatia[1,2] and Laxman Gangwani[1,2,3]*

[1]Center of Emphasis in Neurosciences, Texas Tech University Health Sciences Center, El Paso, TX 79905, USA.

[2]Department of Biomedical Sciences, Paul L. Foster School of Medicine, Texas Tech University Health Sciences Center, El Paso, TX 79905, USA.

[3]Graduate School of Biomedical Sciences, Texas Tech University Health Sciences Center, El Paso, TX 79905, USA.

Contents:

Supplementary Methods

Supplementary Figures (9)

Supplementary Movie (1)

To whom correspondence should be addressed:

Laxman Gangwani
Department of Biomedical Sciences
Paul L. Foster School of Medicine
TTUHSC, El Paso, TX 79905, USA
Email: Laxman.Gangwani@ttuhsc.edu
Phone: (915) 215-4189

SUPPLEMENTARY METHODS

Mouse Skeleton Staining

Mouse skeleton staining was performed using Alcian blue and Alizarin red staining method[1]. Control and mutant E18.5 embryos were collected, skin and internal organs were removed and fixed in 90% ethanol for a week. To stain cartilage, embryos were stained with 0.01% Alcian blue 8GX (Sigma, #S-9640) prepared in ethanol and glacial acetic acid (7:3) for 2 days followed by washing with ethanol/glacial acetic acid solution for 2 h. Embryos were washed with 70% ethanol and double distilled water for 12 h and treated with 1% KOH for 2-3 days until they became clear. To stain mineralized bones, embryos were incubated in 0.001% Alizarin red solution prepared in 0.5% KOH for 2-3 days. Finally, embryos were washed with 1% KOH 5-6 times for several hours each followed by incubations in a gradient series of glycerol (%) in KOH solution (20%, 40%, 60%, 80%), and stained and cleared embryos were stored in 100% glycerol with a piece of thymol crystal. Images were obtained with a digital camera (Canon G12).

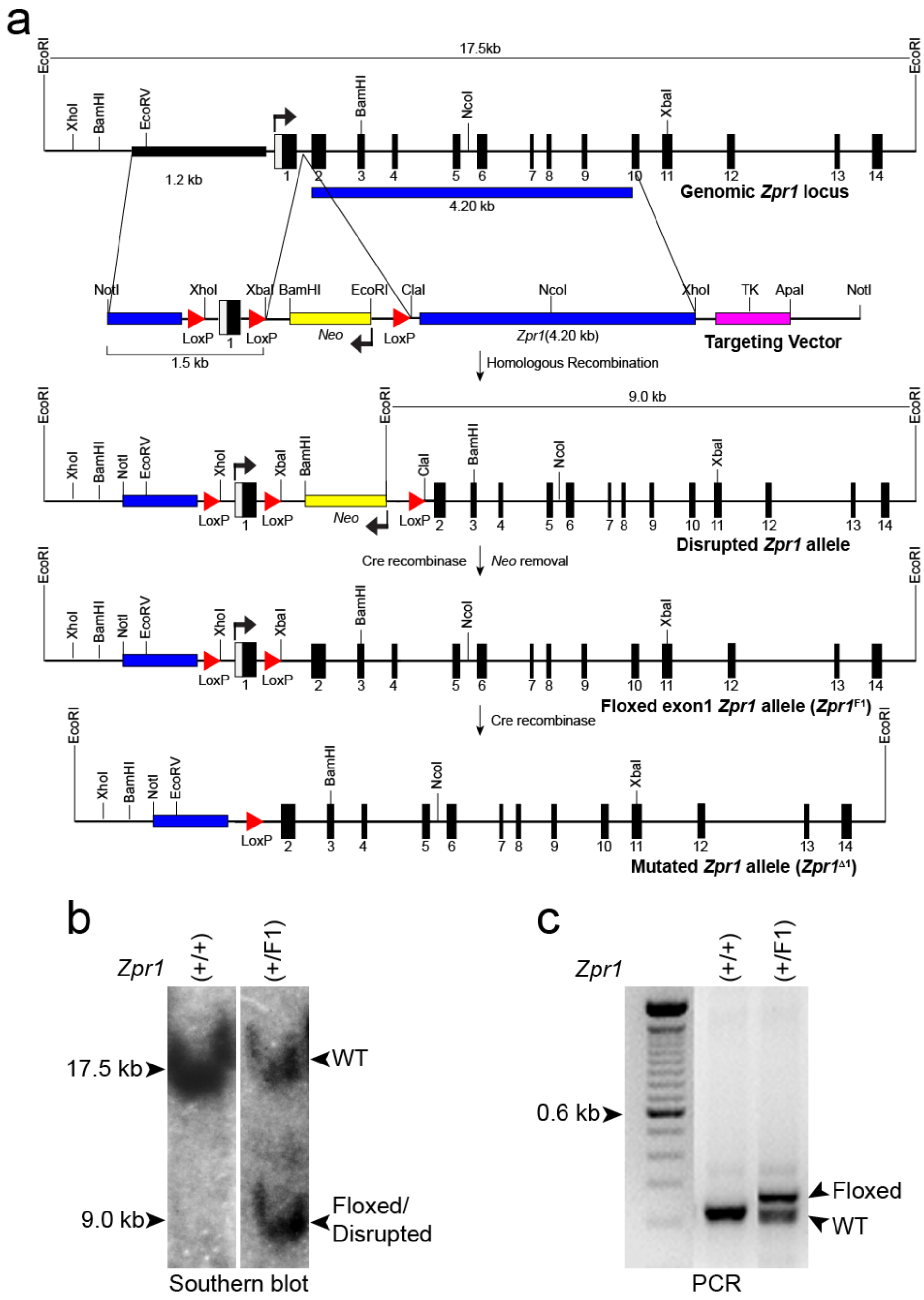

Figure S1. Creation of genetically modified mouse with a conditional allele of the *Zpr1* gene. (a) Strategy used to create mice with a conditional *Zpr1* allele is presented. Murine genomic *Zpr1* locus shown as a line drawing; numbered rectangles represents coding exons. A targeting vector was designed to insert *loxP* sites, flanking *Zpr1* exon 1 and the Neo^R gene in the intron between exon 1 and exon 2. Strategy to generate mice with a floxed exon 1 of *Zpr1* ($Zpr1^{+/F1}$) allele is presented. **(b)** Southern blot analysis of targeted ES cells. Genomic DNA from targeted ES cells was digested with EcoRI and separated by agarose gel electrophoresis and blotted on Zeta Probe membrane. A 425bp cDNA probe spanning exon 10-14 was labeled with ^{32}P and used for Southern blot analysis. Correctly targeted ES cells were used to create chimeric mice. Chimeric mice were crossed to generate germ-line transmission of targeted *Zpr1* allele. **(c)** The floxed Neo^R gene was removed *in vivo* by crossing targeted *Zpr1* allele mice with Protamine3-Cre (*PC3-Cre*) mice to generate mice with floxed exon 1 *Zpr1* allele, $Zpr1^{+/F1}$. Genotypes of mice with floxed allele were examined by PCR using tail DNA from mice. $Zpr1^{+/F1}$ mice were crossbred to generate $Zpr1^{F1/F1}$ mice.

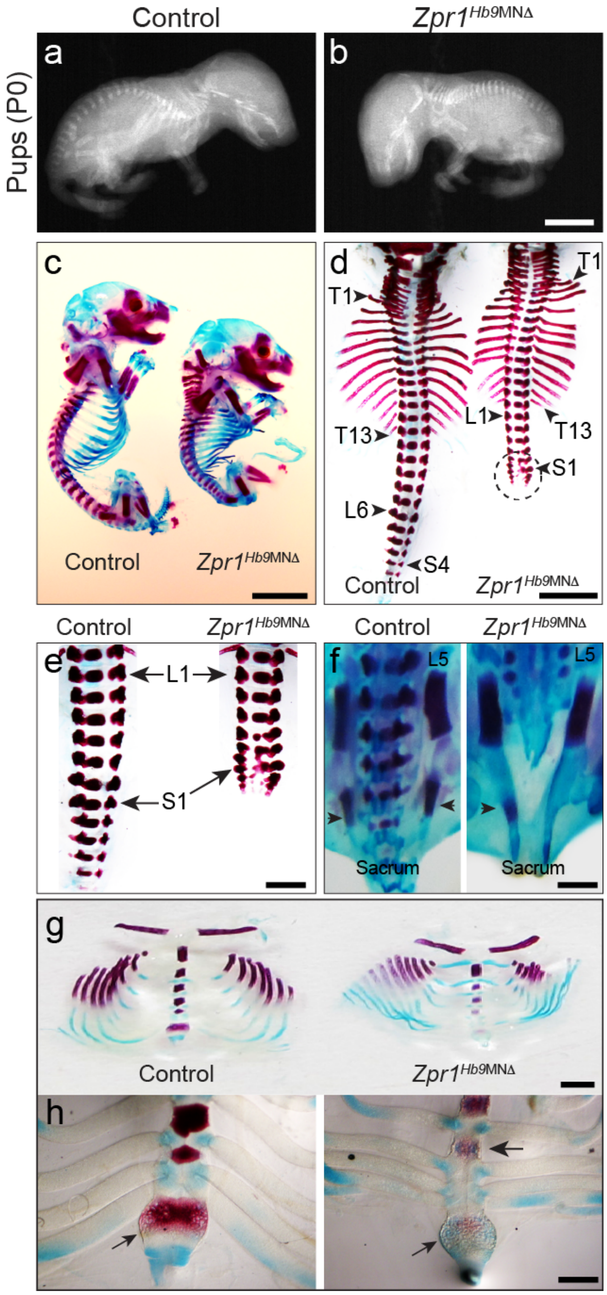

Figure S2. ZPR1-deficiency causes defects in the development of skeleton in *Zpr1*$^{Hb9MN\Delta}$ mice. (a-b) The X-ray radiographs of control and *Zpr1*$^{Hb9MN\Delta}$ mice were examined to determine defects in the skeleton development. Examination of radiographs shows mutant mice skeleton was smaller than control mice suggesting growth defects in mutant mice. Counting of vertebrae showed identical number of vertebrae in the cervical (7), thoracic (13) and lumbar (6) regions of the spinal cord in control and mutant *Zpr1*$^{Hb9MN\Delta}$ mice. Tailbones were absent in mutant *Zpr1*$^{Hb9MN\Delta}$ mice. **(c-h)** Embryos skeletons were stained with Alcian blue (cartilage) and Alizarin red (mineralized bones). **(c)** Whole embryo staining shows mutant *Zpr1*$^{Hb9MN\Delta}$ embryo has overall smaller size with larger head compared to control embryo. **(d)** Comparison of ribs and vertebral columns shows defects in the development of sacral region, underdeveloped and fused sacral vertebrae in mutant embryo (dotted circle), **(e)** enlarged images of sacral region vertebrae, **(f)** defects in the development of sacrum, including absence of ischium bone on one side (bones indicated by arrowheads), **(g)** smaller sternum in mutant embryo and **(h)** reduced ossification of lower sternebrae in mutant embryos. Scale bars are 5 mm (a, b, c), 2.5 mm (d), 1 mm (e, f, g) and 0.5 mm (h).

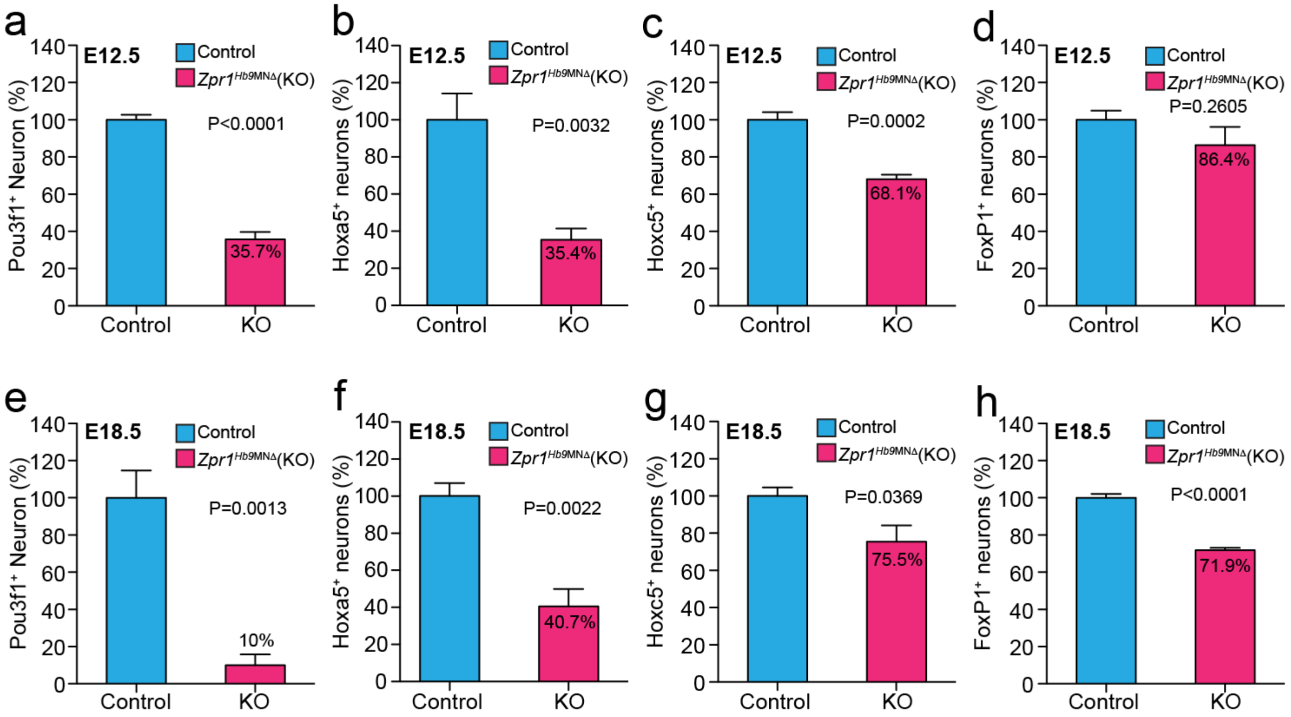

Figure S3. Quantitative analysis shows progressive loss of PMC neurons in the cervical region of the spinal cord in *Zpr1*$^{Hb9\text{MN}\Delta}$ mice. (a-d) Quantification of Pou3f1^{+}, Hoxa5^{+}, Hoxc5^{+}, and FoxP1^{+} neurons at E12.5 and **(e-h)** E18.5 stages is presented as bar graphs. Neurons were stained with antibodies and counted in serial sections of C3-C5 region of spinal cords from control and *Zpr1*$^{Hb9\text{MN}\Delta}$ mice. Neuron numbers are presented as relative % of neurons (mean ± SEM; n = 3 mice/group). At E12.5 stage, quantification shows loss (64.29 ± 4.78%, p = 0.000) of Pou3f1^{+} motor neurons. By E18.5 stage Pou3f1^{+} neurons were barely detectable and quantification shows greater loss (90.00 ± 15.81%, p = 0.001) of neurons in *Zpr1*$^{Hb9\text{MN}\Delta}$ mice. Quantification of Hoxa5^{+} neurons shows non-progressive loss of neurons at E12.5 (64.64 ± 15.54%, p = 0.003) and at E18.5 (59.35 ± 11.60%, p = 0.002) stages in *Zpr1*$^{Hb9\text{MN}\Delta}$ embryos. A smaller loss of Hoxc5^{+} neurons at E12.5 (31.93 ± 4.80%, p = 0.000) and E18.5 (24.51 ± 9.81%, p = 0.036) stages was noted in *Zpr1*$^{Hb9\text{MN}\Delta}$ mice. A relatively smallest decrease in FoxP1^{+} LMC neurons at E12.5 (13.59 ± 10.94%, p = 0.260) that increases to a statistically significant loss at E18.5 (28.17 ± 2.42%, p = 0.000) was found in *Zpr1*$^{Hb9\text{MN}\Delta}$ mice.

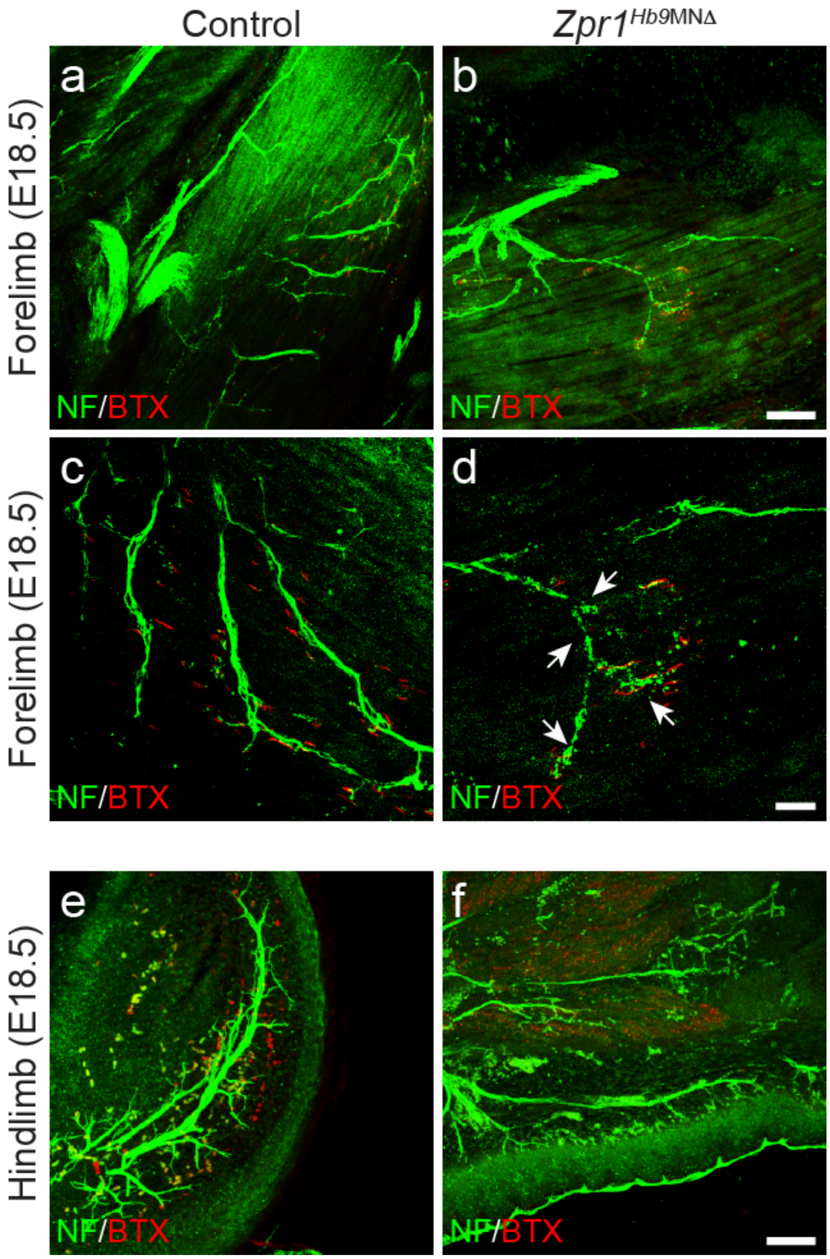

Figure S4. Mutation of *Zpr1* in motor neurons causes nerve innervation defects in limbs of $Zpr1^{Hb9\text{MN}\Delta}$ mice. (a-d) Forelimbs and **(e-f)** hindlimbs of E18.5 control and $Zpr1^{Hb9\text{MN}\Delta}$ embryos were stained with neurofilament (NF) antibody and α-Bungarotoxin (BTX). Immunofluorescence analysis revealed defects in the innervation of nerves and the formation of neuromuscular junctions (NMJs) in $Zpr1^{Hb9\text{MN}\Delta}$ mice. **(c-d)** Higher magnification of innervated muscle in the forelimb shows degeneration of secondary and terminal branches of nerves (arrows) suggesting that ZPR1 is required for the maintenance of motor neurons in the nerve. Scale bars are 200 μm (a, b), 25 μm (c, d) and 100 μm (e, f).

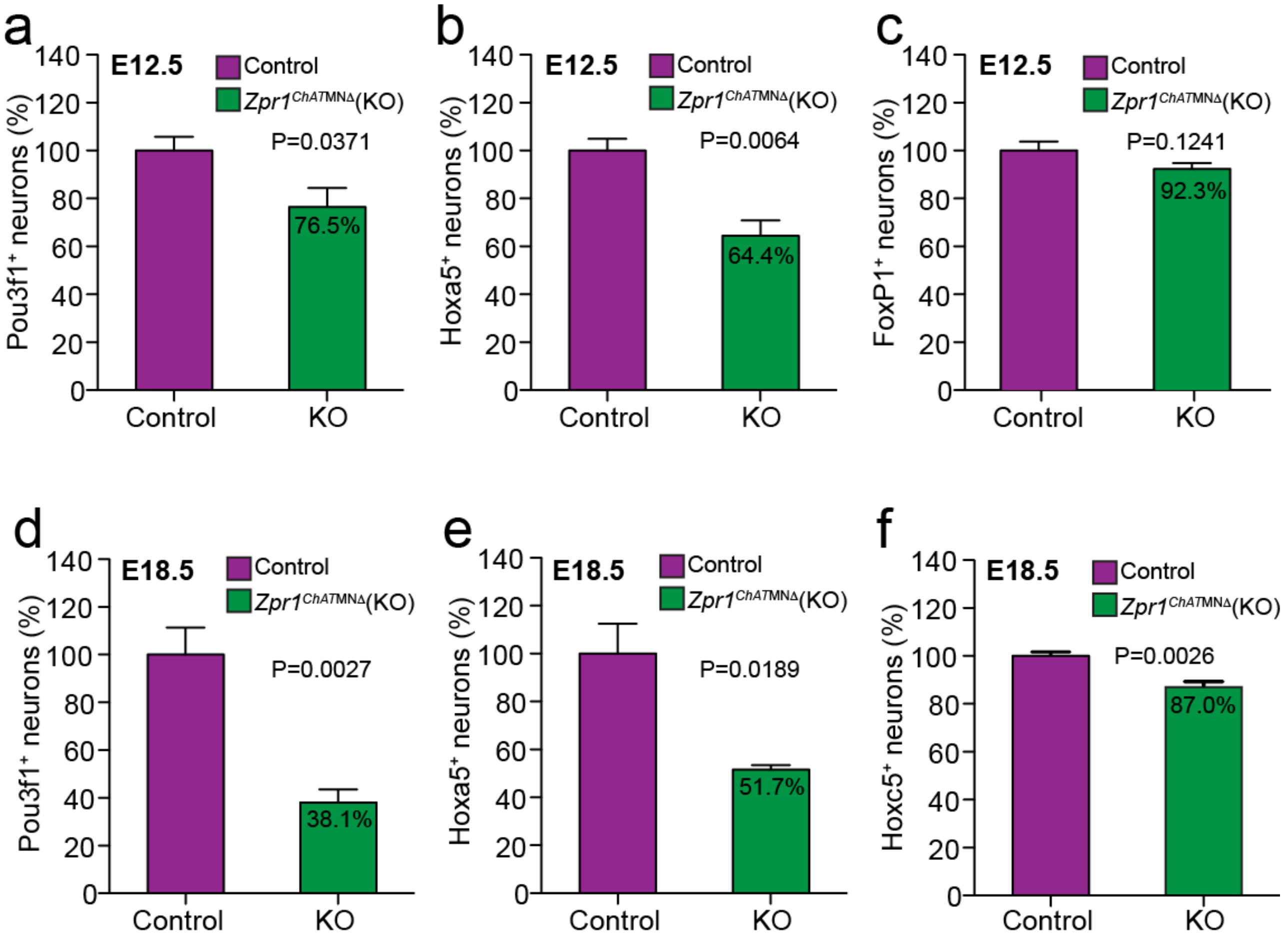

Figure S5. Quantitative analysis shows progressive loss of PMC neurons in the cervical region of the spinal cord in *Zpr1*$^{ChAT\text{MN}\Delta}$ mice. Neurons were stained with antibodies and counted in serial sections of C3-C5 region of the spinal cords from control and *Zpr1*$^{ChAT\text{MN}\Delta}$ mice. Quantification of Pou3f1$^+$, Hoxa5$^+$ and Hoxc5$^+$ neurons in the cervical C3-C5 region of spinal cords from control and *Zpr1*$^{ChAT\text{MN}\Delta}$ E12.5 and E18.5 embryos are presented as relative % of neurons (mean ± SEM; n = 3 mice/group) in bar graphs. **(a-c)** Quantification of neurons in E12.5 embryo spinal cords shows the loss of Pou3f1$^+$ (23.50 ± 9.77%, p = 0.0371) and Hoxa5$^+$ (35.56 ± 7.89%, p = 0.0064) neurons in *Zpr1*$^{ChAT\text{MN}\Delta}$ embryos. The loss of FoxP1$^+$ (7.70 ± 7.89%, p = 0.1241) neurons in E12.5 *Zpr1*$^{ChAT\text{MN}\Delta}$ embryos was not significant. **(d-f)** Quantification of neurons in E18.5 embryo spinal cords shows loss of Pou3f1$^+$ (61.90 ± 12.59%, p = 0.002) PMC motor neurons in *Zpr1*$^{ChAT\text{MN}\Delta}$ mice. Quantification of Hoxa5$^+$ and Hoxc5$^+$neurons shows loss of (48.34 ± 12.67%, p = 0.018) and (12.97 ± 1.93%, p = 0.002), respectively.

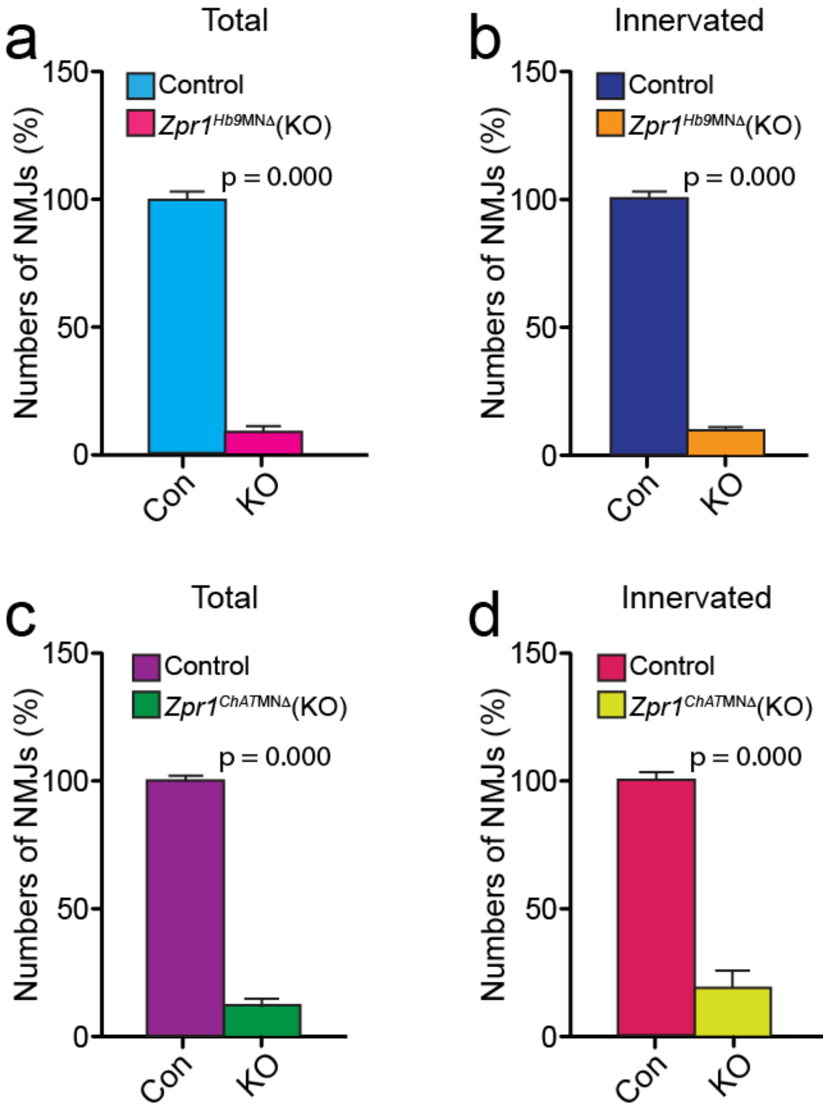

Figure S6. Quantification of neuromuscular junctions (NMJs) in the diaphragms of control and mutant, *$Zpr1^{Hb9\text{MN}\Delta}$* and *$Zpr1^{ChAT\text{MN}\Delta}$* mice. Diaphragms from E18.5 control and *$Zpr1^{Hb9\text{MN}\Delta}$* and *$Zpr1^{ChAT\text{MN}\Delta}$* mice stained with neurofilament antibody and α-bungarotoxin (BTX) used for counting of total NMJs and innervated NMJs as presented in main Figures 2 and 6. Quantification is presented as relative % of NMJs (mean ± s.e.m.; n = 4 mice/group) as bar graphs. **(a)** Total NMJs in Control (100.3 ± 2.210)% and *$Zpr1^{Hb9\text{MN}\Delta}$* (9.883 ± 1.343)% mice, **(b)** innervated NMJs in Control (100.3 ± 2.724)% and *$Zpr1^{Hb9\text{MN}\Delta}$* (9.560 ± 0.7942)% mice, **(c)** total NMJs in Control (100.0±1.612)% and *$Zpr1^{ChAT\text{MN}\Delta}$* (12.92 ± 2.049)% mice and **(d)** innervated NMJs in Control (100.0 ± 3.292)% and *$Zpr1^{ChAT\text{MN}\Delta}$* (19.44 ± 6.012)% mice. Statistical significance (p value) was determined by unpaired t-test.

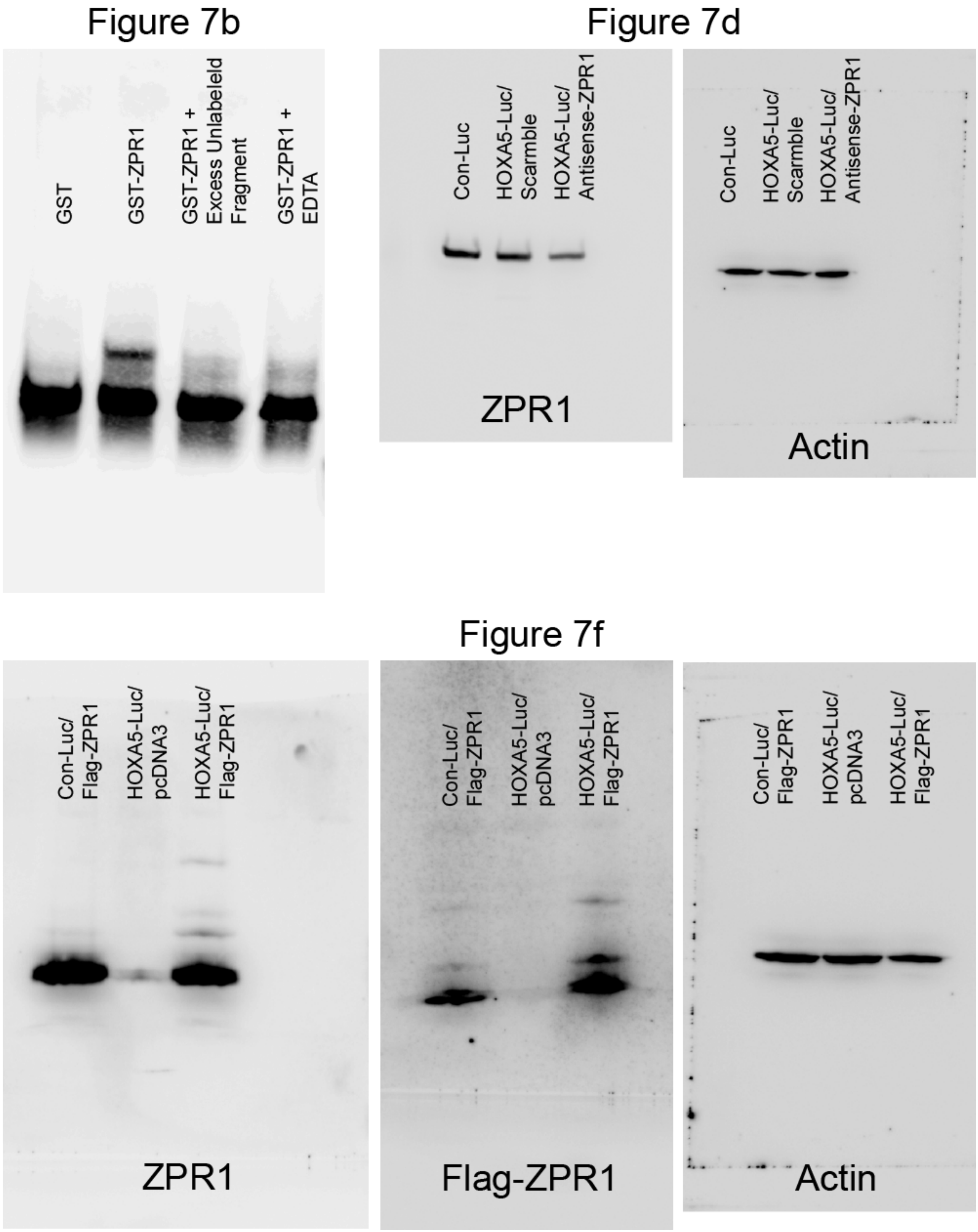

Figure S7. Full-length blots of cropped images presented in main manuscript Figure 7.

Figure 8a

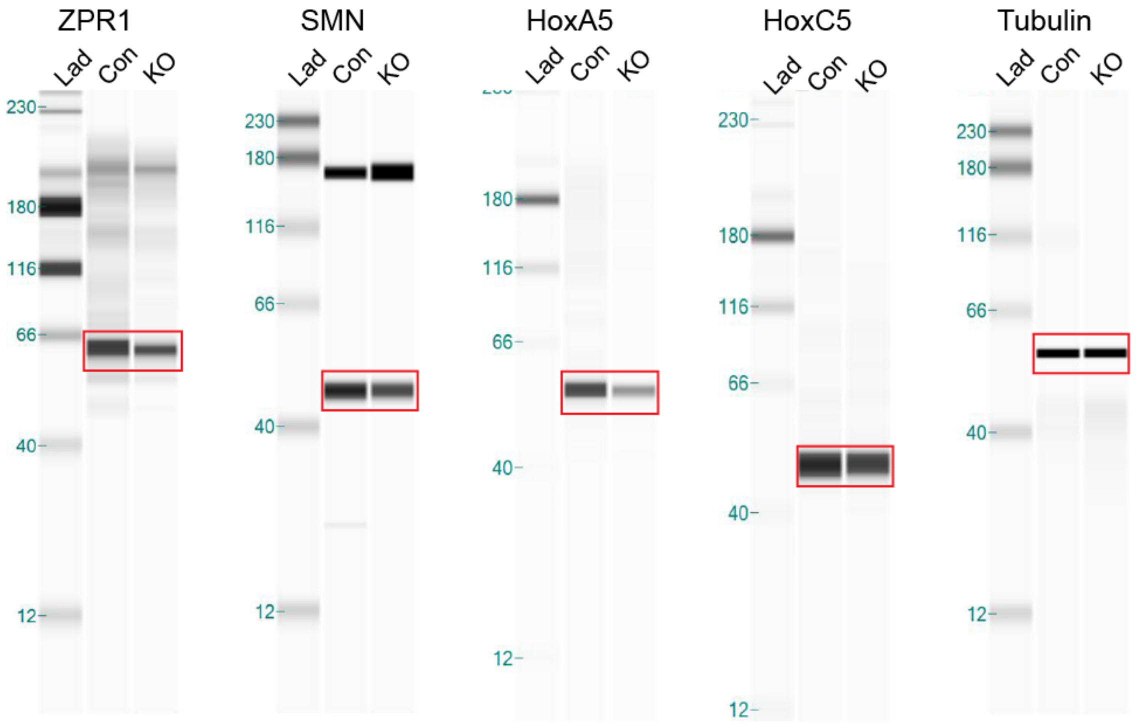

Figure 8b

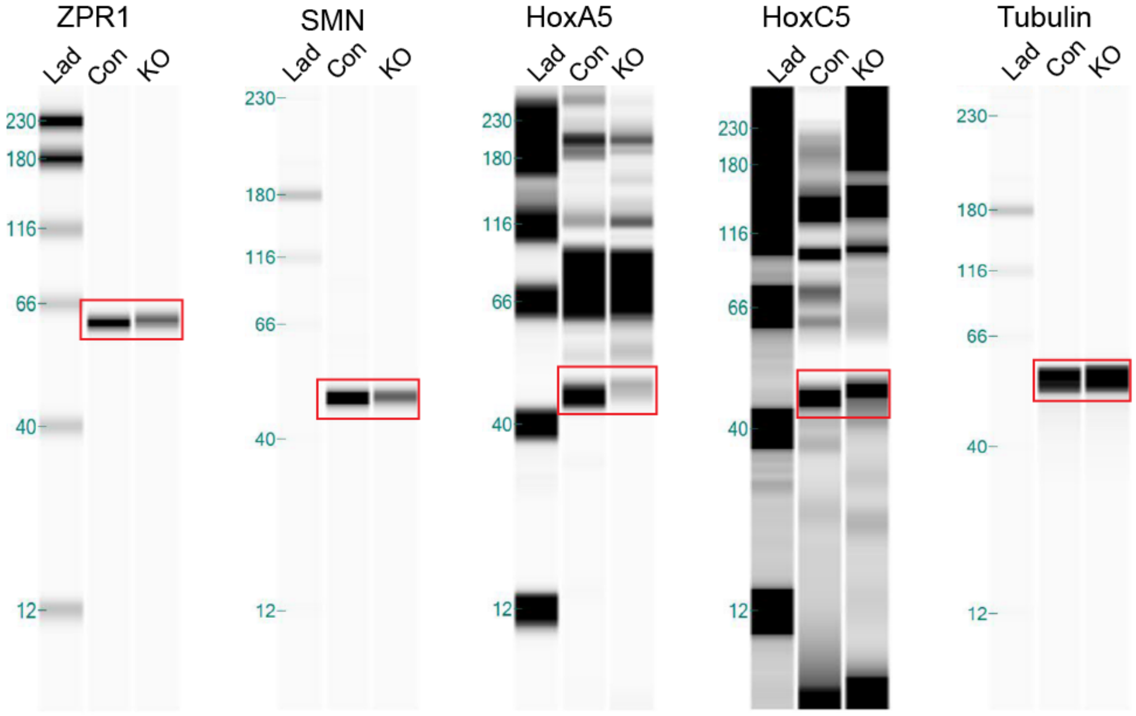

Figure S8. Full-length blots of cropped images presented in manuscript Figure 8a and 8b. Gel bands cropped are highlighted with red color box.

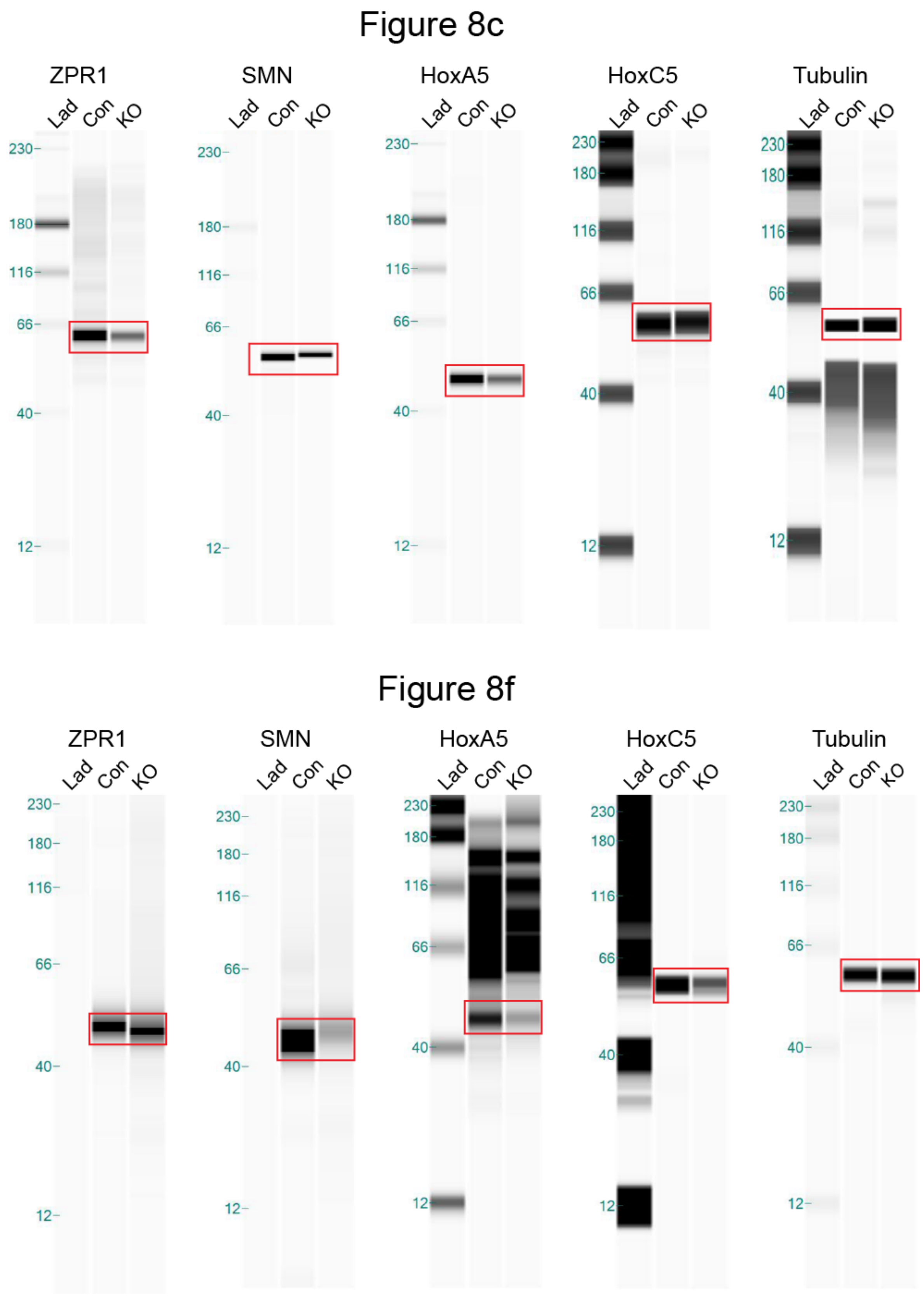

Figure S9. Full-length blots of cropped images presented in manuscript Figure 8c and 8f. Gel bands cropped are highlighted with red color box.

Movie S1. Mutation of the *Zpr1* gene in the spinal cord motor neurons causes respiratory failure in mice. Analysis of respiration in E18.5 control and mutant $Zpr1^{Hb9\mathrm{MN}\Delta}$ embryos. Video shows respiration attempts by E18.5 littermates. Top row, control embryos with tail and bottom row, mutant embryos without tail. Control embryos initiated breathing successfully. One of the mutant embryos did not make any attempt for breathing; other mutant embryo made attempt to initiate breathing but failed to sustain breathing and died.

REFRENCES

1 Zhang, P. *et al*. The PERK eukaryotic initiation factor 2 alpha kinase is required for the development of the skeletal system, postnatal growth, and the function and viability of the pancreas. *Mol Cell Biol* **22**, 3864-3874 (2002).
